# CAU-10-H: Synthesis Scale-Up at the Pilot Scale, Techno-Economic Analysis, and Application in a Full-Scale Cooling System

**DOI:** 10.1021/acs.iecr.5c05308

**Published:** 2026-03-26

**Authors:** Kalle S. Mertin, Abeer Mohtar, Marta Bordonhos, Moisés L. Pinto, Thomas May, Ralph Herrmann, Norbert Stock

**Affiliations:** † Institut Für Anorganische Chemie, 9179 Christian-Albrechts-Universität zu Kiel, Kiel 24118, Germany; ‡ CERENA, Departamento de Engenharia Química, Instituto Superior Técnico, 72971 Universidade de Lisboa, Av. Rovisco Pais 1, Lisboa 1049-001, Portugal; § CICECO—Aveiro Institute of Materials, Department of Chemistry, University of Aveiro, Campus Universitário de Santiago, Aveiro 3810-193, Portugal; ∥ SorCool GmbH, Zscherbener Landstraße 12, Halle 06126, Germany; ⊥ Kiel Nano, Surface and Interface Science KiNSIS, Christian-Albrechts-Universität zu Kiel, Kiel 24118, Germany

## Abstract

Metal–organic frameworks (MOFs) offer a wide range of advantages for modern society. In particular, the growing societal challenges of energy consumption for cooling and water scarcity can be addressed by high-performance MOFs, such as CAU-10-H, Al-MIL-160, and MOF-303. To enable the application of MOFs, sustainable and large-scale production must be established. Here, we report the green, low-cost multikilogram scale-up of CAU-10-H in a pilot-scale batch reactor leading to 27.5 kg of dry MOF, corresponding to a space–time yield (STY) of 99 kg m^–3^ d^–1^. Based on the results, a techno-economic analysis was performed to estimate the production cost for a production process at a 1 kt scale. The scenario considered achieved a cost of 13.8 ± 2.8 $ kg^–1^ (2022 prices). Linker costs were identified as the main cost-driving parameter. Further optimization of the reaction conditions was performed on a 10 L scale, including solvent recycling and increased reaction concentration by a factor of 2. The latter resulted in a projected STY above 400 kg m^–3^ d^–1^ without compromising the MOF properties. The best-case scenario (STY = 481 kg m^–3^ d^–1^) leads to a reduction in production cost to 12.1 ± 2.4 $ kg^–1^. Water adsorption capacities and rates of CAU-10-H coatings were measured to evaluate their use in adsorption cooling (ADC). An adsorption chiller simulation showed clear benefits in cooling efficiency for air conditioning in moderate climates and engine heat-driven ship cooling by a factor of 3 against silica gel and a factor of 2 against SAPO-34, the current state-of the art material.

## Graphical abstract

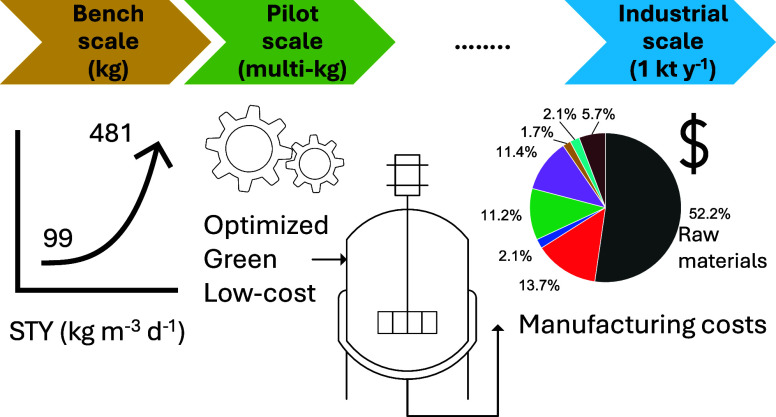

## 1 Introduction

Microporous adsorbents composed of metal ions and coordinating bridging organic linkers, named metal–organic frameworks (MOFs), have gained increased interest in academic and industrial research in recent years due to their potential to tailor material properties to the desired application. This year’s Nobel Prize in Chemistry, awarded for the discovery and development of these materials, underlines their potential.^1^ The possible applications for MOFs range, for example, from gas separation and storage to sensing, heterogeneous catalysis, and drug delivery.^2–5^ However, only a few MOFs have found their way into real-world applications.^6,7^ This is due to the limited amount of companies working on the production and commercialization of MOF materials. Most of the companies producing MOFs are start-ups that specialize in one or a few MOF materials, resulting in the scarce commercial availability of MOFs on a kilogram or tonne scale (Table S1),^8^ with selected MOFs currently costing several hundred dollars for a few grams and, if available in bulk, often exceeding several thousand dollars per kilogram. Hence, MOFs have not found their way into the global market and large-scale applications yet. Some exemplary quotes are given in Table S2.^9–12^

Several synthetic routes and techniques have been reported for obtaining MOF materials, most of which are not suitable for effective scale-up due to safety risks of the employed reactants or solvents, necessity of applying elevated pressure, or high-energy consumption. These include the classical solvothermal, electrochemical, or continuous flow syntheses in plug flow reactors.^13^ The most promising synthesis methods for industrial MOF production are those carried out in aqueous solutions at ambient pressure or principally solvent-free reactions, *i.e.*, mechanochemical syntheses.^8,14^ Very recently, a very efficient spray drying process was reported for the scale-up of aluminum fumarate, a MOF that is easily obtained from aqueous solution.^15^

In order to make MOFs available at low cost and thus exploit their attractive properties for real-world large-scale applications, it is important to establish green, sustainable synthetic routes using nontoxic, low-cost reactants that are scalable from the laboratory to industrial scale and can be performed with high space–time yields (STY).^7,8,13^ To evaluate MOF production, techno-economic analyses must be carried out to estimate a production price. These can also be used to identify the respective drivers for further optimization. Techno-economic analyses of several MOF materials have recently been published.^16–18^ These include Fe-MIL-100, Al-MIL-120, and Al-MIL-160, and based on an annual production scale of 1 kt, prices have been estimated to be as low as 30 $ kg^–1^, 13 $ kg^–1^, and 29.5 $ kg^–1^, respectively, with the final production cost greatly influenced by the cost of raw materials/reactants.

A promising MOF for application in water-adsorption-based technologies, such as adsorption-driven cooling (ADC) or atmospheric water harvesting (AWH), is CAU-10-H.^19–22^ Discovered over 10 years ago by Reinsch et al.,^23^ it has been extensively studied and tested for application in ADC, and its synthesis has been optimized to a green and sustainable route.^21,23,24^ The MOF exhibits a steep one-step uptake of ∼30 wt % water vapor at around 17% RH, which has been demonstrated to be fully reversible for at least 10 000 cycles under working conditions, with a low regeneration temperature of 70 °C.^24^ The water adsorption properties of CAU-10-H have been characterized most thoroughly.^19,24^ Moreover, in the past few years, there have been a large number of adsorption and separation studies carried out on pure CAU-10-H, its composites, or derivatives containing additional functional groups. There is a more detailed evaluation on the sorption properties of CAU-10-H given in the Supporting Information Section S1, Table S3. These studies highlight further potential applications of CAU-10-H in areas such as natural gas purification, bioethanol steam reforming, separation of MeOH/MTBE from azeotropic liquid, or flue gas desulfurization.^25–28^

An important step toward the commercialization of CAU-10-H was the 10 L laboratory-scale, water-based synthesis using commodity chemicals under reflux conditions with high yields of 90–95%.^21^ Additionally, purification and activation can be carried out under mild conditions, *i*.*e*., more efficiently than for most other MOFs, which often require additional processing steps such as solvent exchange and treatment under vacuum at elevated temperatures.^29,30^ Recently, the synthesis of several MOFs has been scaled up, including CAU-10-H, using a 50 L reaction volume with an STY of 305 kg m^–3^ d^–1^.^31^ The STY values reported in the literature are an important indicator of the efficiency of scale-up, but one has to keep in mind that many parameters contribute to the STY values of the complete production process. For example, the time necessary for the preparation of starting materials, transfer, heating to reaction temperature, reaction, cooling of the reaction mixture, isolation of the product, purification, and drying are rarely reported in this detail. The reported STY of 305 kg m^–3^ d^–1^ was calculated by considering only the synthesis time and would significantly decrease if the entire process is considered. STY values of other industrially produced MOFs, like for BASF’s Basolite series, have been reported to range from 20 to >300 kg m^–3^ d^–1^.^32^

MOFs can act as core components in adsorption-based cooling systems.^33^ Their large inner surface can be modified to have tailored hydrophilic properties to use waste heat temperatures of 50–95 °C for desorption and still adsorb water above 40 °C ambient temperature. These properties enable adsorption cooling with low grade heat coming from common sources like combustion engines in cars, ships, or generators, industrial processes, high power computation, electrolysis, or solar panels and lead to adsorption chillers using only a small fraction of the electric power of conventional compression chillers. To highlight CAU-10-H feasibility for such applications, there is a comparison of the production and activation procedure of other promising MOFs for H_2_O adsorption given in the Supporting Information Section S1, Table S4.

With the increasing global cooling demand that may double its 2020 electricity consumption until 2050 to 16 000 TWh, there is a strong need to have energy efficient technologies like thermal cooling available.^34,35^ CAU-10-H has the potential to promote solar or waste heat-driven adsorption cooling since waste heat in the EU region has a capacity of 920 TWh, often offering energy from industrial processes where cooling is required nearby. This presents optimal conditions for adsorption cooling by making it more effective, efficient, and economically viable.^36^ If only 0.2% of the European industrial waste heat could be used for CAU-10-H adsorption cooling, around 1 000 t of the MOF would be required. Therefore, very large amounts (multitonne scale) of the adsorbent would be required to make use of at least a fraction of the waste heat. On the other hand, a 1 000 t CAU-10-H production could enable air conditioning in moderate climates in 13 680 000 m^2^, which counts for 171 000 apartments of 80 m^2^, where private housing only very rarely has required heat sources (like solar-thermal panels) available. There is a more detailed discussion about the potential electricity savings and the expected demand of CAU-10-H given in the Supporting Information Section S6.

Here, we report on the multikilogram scale-up of CAU-10-H using pilot scale reactors and its techno-economic analysis on two production scales to assess its commercialization potential in different applications, especially the benefits of using it in adsorption-driven cooling.

## 2 Experimental Section

### 2.1 Materials and Methods

All chemicals were obtained commercially and used without further purification: sodium hydroxide (NaOH, Bienenzuchtbedarf Geller GmbH, 99.5%), isophthalic acid (*m*-H_2_BDC, fisher scientific, 99%), aluminum sulfate octadecahydrate (Al_2_(SO_4_)_3_·18 H_2_O, Fisher Scientific, ≥98%), sodium aluminate (NaAlO_2_, Sigma-Aldrich, technical), ethanol (C_2_H_5_OH, Stockmeier Group, 96%, 1 vol % MEK), and SilRes MP50E (Wacker Chemie AG). Seeding crystals of CAU-10-H were obtained by synthesis in the lab-scale 10 L reaction setup.^21^ For the scale-up investigations, the amount of chemicals was measured using a Ravas RPW-2100EXI pallet scale with a maximum load of 2 200 kg. Product yield was determined by using a Sartorius 50 kg scale. The scaled-up synthesis was carried out in a stirred 750 L stainless-steel reactor by Schwarz Apparatebau. Product separation was carried out using a heated Seitz TERRA EF60/125CW stirred batch filter under excess pressure in a nitrogen atmosphere with a Begerow BECO-CP 00 type filter paper (1.5–17 μm, diameter 65 cm, height 4 mm). The product was dried in a conventional convection oven at 90 °C for 24 h.

The characterization of the products was carried out by powder X-ray diffraction (PXRD), volumetric N_2_ and gravimetric H_2_O sorption measurements, thermogravimetric and differential thermal analysis (TGA/DTA), elemental analysis (EA) for carbon, hydrogen, nitrogen, and sulfur, static light scattering (SLS), and infrared (MIR-) and Raman spectroscopy. PXRD measurements were performed on a Stoe Stadi P instrument with a MYTHEN2 1K detector (Cu Kα_1_-radiation, λ = 1.5406 Å). The volumetric N_2_ sorption experiments were carried out on a BelSorp Max instrument by Microtrac at 77 K. Before the measurement, the sample was activated for 16 h at 180 °C under reduced pressure. The apparent specific surface area and the micropore volume (at p p_0_^–1^ = 0.5) was calculated using BELMaster 7.0 software using the Rouquerol approach.^37^ The gravimetric H_2_O sorption isotherms were recorded on a DVS Advantage by Surface Measurement Systems at 298 K. Before the measurement, the sample was activated by purging in a dry nitrogen atmosphere (200 cm^3^ min^–1^) for 3 h at 200 °C. TGA was performed on a Linseis STA 1600 up to 700 °C with a heating rate of 8 K min^–1^ in air with a flow rate of 6 L h^–1^. EA was carried out with a vario MICRO cube elemental analyzer from Elementar Analysensysteme GmbH. SLS analysis was performed with a HORIBA LA-950 V2 particle size analyzer. IR spectra were recorded on a Bruker ALPHA-P ATR MIR spectrometer. Raman spectra were recorded at room temperature on a Bruker RAM II FT-Raman spectrometer using a liquid nitrogen cooled highly sensitive Ge detector and 1064 nm laser radiation.

### 2.2 Synthesis in the 750 L Batch Reactor

#### 2.2.1 Preparation of Starting Solutions

The optimized green synthesis of CAU-10-H in the 10 L reactor, as reported in a previous work, uses a molar ratio of Al­(III): *m*-H_2_BDC:H_2_O: C_2_H_5_OH = 1:1:902:7.3, resulting in a total volume of 8.552 L of the reaction mixture. More details are given in the Supporting Information, Section S2, Table S5.

For the synthesis in the 750 L reactor, the same molar ratios were used and the synthesis volume was increased by a factor of 60. For **solution 1**, 12.00 kg (300.02 mol) of NaOH was dissolved in 150 kg of deionized water in a stirred 1000 L IBC (intermediate bulk container). 24.90 kg (149.87 mol) of *m*-H_2_BDC was added to the solution, and the mixture was stirred for 30 min. After the initial homogenization period, 150 kg of deionized water was added, and the solution was stirred for another 2 h. For **solution 2**, 37.90 kg (56.871 mol) of Al_2_(SO_4_)_3_·18 H_2_O was added to 112.5 kg of deionized water in a 205 L steel barrel with a PE-inlet. In a separate 205 L steel barrel with a PE-inlet, 3.070 kg (37.452 mol) of NaAlO_2_ was added to 75.00 kg of deionized water as **solution 3**. **Solution 1** and 20.30 kg of ethanol were transferred to the 750 L reactor using negative pressure (600 mbar) within 65 min under stirring at 100 rpm and left overnight. Dissolution of the metal salts turned out to be time-consuming, and therefore, **solutions 2** and **3** were left overnight to ensure complete dissolution.

#### 2.2.2 Transfer and Homogenization

The synthesis was started by transferring **solution 2** into the reactor using a negative pressure (800 mbar) over a period of 80 min. The formed precipitate increases the viscosity of the mixture, so the mixture was homogenized at 100 rpm for 1.5 h. Subsequently, **solution 3** was transferred to the reactor using negative pressure (800 mbar) for 83 min. Ultimately, 195.0 g of CAU-10-H seed crystals were added to the reactor. The reaction mixture was homogenized by stirring for 30 min.

#### 2.2.3 Heating and Synthesis

After homogenization, heating the rack to the target temperature of 120 °C was carried out in several steps to prevent rapid expansion within the heating rack, which could potentially damage the system. The final temperature of the heating rack was reached after 1.5 h. The reaction temperature of 95 °C of the reaction mixture was reached after 4 h 50 min, after which the synthesis was continued isothermally for 14 h overnight.

#### 2.2.4 Product Isolation and Washing

The reaction was stopped by closing the valve to the condenser and pressurizing the sealed reactor to 2–2.5 bar with a N_2_ atmosphere. The heating mantle of the batch filter was set to 120 °C, and the product was transferred within 1 h 23 min. The filter cake was first washed with 180 kg of hot deionized water (100 °C) while being stirred, followed by another 100 kg of hot deionized water for a second washing step. The washing was finished after 2 h and 6 min. At the beginning of the filtration, a rupture of the filter paper led to a loss of around 50 L of the reaction mixture. By using Begerow BECO-CP 00 type filter paper, the product was isolated from the rest of the reaction mixture without any problems.

#### 2.2.5 Drying

After cooling to ambient temperature overnight, the product was transferred to several stainless-steel containers, covered with perforated aluminum foil to prevent dust formation, and placed in a drying oven at 90 °C for 24 h.

A photographic documentation of the process and setup is given in the Supporting Information Section S2, Figures S1 to S5.

### 2.3 Synthesis Optimization Studies to Improve STY Values

Laboratory-scale experiments were carried out to further improve the STY of the synthesis of CAU-10-H. A 10 L glass reactor with an oil heating jacket was used. The concentration of the reactants was doubled, and the recycling of the solvent was investigated in four consecutive runs. Therefore, the filtrate of the mother liquor was cooled to room temperature, and the precipitated isophthalic acid was removed by filtration. The remaining solution was used again for the next synthesis, with water and ethanol added in the required amount. The molar ratios of the starting materials are given in the Supporting Information Table S5, and a detailed description of the synthesis conditions and full product characterization are given in the Supporting Information, Section S5.

## 3 Results and Discussion

The synthesis of CAU-10-H was successfully scaled up from a 10 L to a 750 L reactor using identical molar ratios by increasing the synthesis volume by a factor of 60 from 8.55 to 513 L. The products and processes carried out in the 10 and 750 L reactors are hereafter referred to as R-10 and R-750, respectively. For ease of understanding Table 1 shows the systematic behind the sample codes used in the main text and the Supporting Information. Crystal seeds of CAU-10-H were added before the heating and process parameters had to be adapted to the available infrastructure. Logically, the production process R-750 takes much longer than laboratory synthesis R-10, which applies to all process steps, *i*.*e*., preparation of starting solutions, transfer and homogenization, heating and synthesis, product isolation, and washing and drying. The process data of CAU-10-H from synthesis in the 750 L reactor (R-750) are presented in Section 3.1. A time comparison of the individual process steps on different synthesis scales is presented and discussed in Section 3.1.1 to identify the parameters for further process optimization. The characterization of CAU-10-H from the 750 L synthesis scale (R-750) is compared with a reference from the 10 L laboratory-scale synthesis (R-10) in Section 3.1.2. The process parameters were used in a techno-economic analysis for the production of 1 kt y^–1^ CAU-10-H, and critical parameters for decreasing the production cost were identified (Section 3.2.). Further optimization of the reaction conditions at a 10 L laboratory scale to improve STY and thus decrease costs is presented in Section 3.3, and the results on the application of CAU-10-H in ADC are presented in Section 3.4.

**Table 1:** Systematic behind the Sample Codes^a^,^b^

	meaning of		
sample code	prefix	number	suffix
R-10	reaction product	10 L reactor	-
R-200	reaction product	200 L reactor	-
R-750	reaction product	750 L reactor	-
R-10-R0	reaction product	10 L reactor	fresh solvent
R-10-R1	reaction product	10 L reactor	1 time recycled solvent
R-10-R2	reaction product	10 L reactor	2 times recycled solvent
R-10-R3	reaction product	10 L reactor	3 times recycled solvent
R-10-IC1	reaction product	10 L reactor	increased concentration 1 mol L^–1^
C1	MOF coating	coating 1	-
C2	MOF coating	coating 2	-

a The list of reactions (R) and coatings (C) is given as the prefix.

b The number describes the scale of the reactor or the number of the coatings (two different coating thicknesses), and the suffix contains additional information on the variations in the synthesis.

### 3.1 Synthesis of CAU-10-H in a 750 L Reactor (R-750)

A total of 27.5 kg dry CAU-10-H was obtained from the scale-up synthesis. With a reaction time of 14 h and a reaction volume of 513 L, this corresponds to a yield of *ca*. 88% and an STY of 92 kg m^–3^ d^–1^. Taking the loss of 50 L of the reaction mixture during the filtration into account, an STY value of 99 kg m^–3^ d^–1^, corresponding to a yield of 95% (29.6 kg), can be extrapolated, which is within the expected range determined in previous studies and demonstrates the robustness of the synthesis procedure (Supporting Information, Table S5). Comparison to other scale-up reactions shows that there is room for improvement.^13^ The energy consumption for heating, synthesis, filtration, and washing was also determined. In total, 569.58 kWh were used, corresponding to an energy cost of 135.84 € (∼147 $), which is comparatively low, so other factors will remain more important for future scale-up investigations (see Sections 3.1.1. and 3.3).

#### 3.1.1 Process Setup

The P&ID scheme of the whole process setup as well as a photograph of the reactor, reflux condenser, and batch filter used for R-750 is given in Figure 1. The containers with **solutions 1**, **2,** and **3** and ethanol, the cosolvent, are shown on the bottom left of the P&ID scheme. The solutions and ethanol were transferred under reduced pressure to the 750 L reactor presented in the middle, which is equipped with a mechanical stirrer and a condenser. Isolation and washing of the final product were carried out in a heated and stirred batch filter shown on the right of the P&ID scheme (blue box in the photograph).

**Figure 1:**
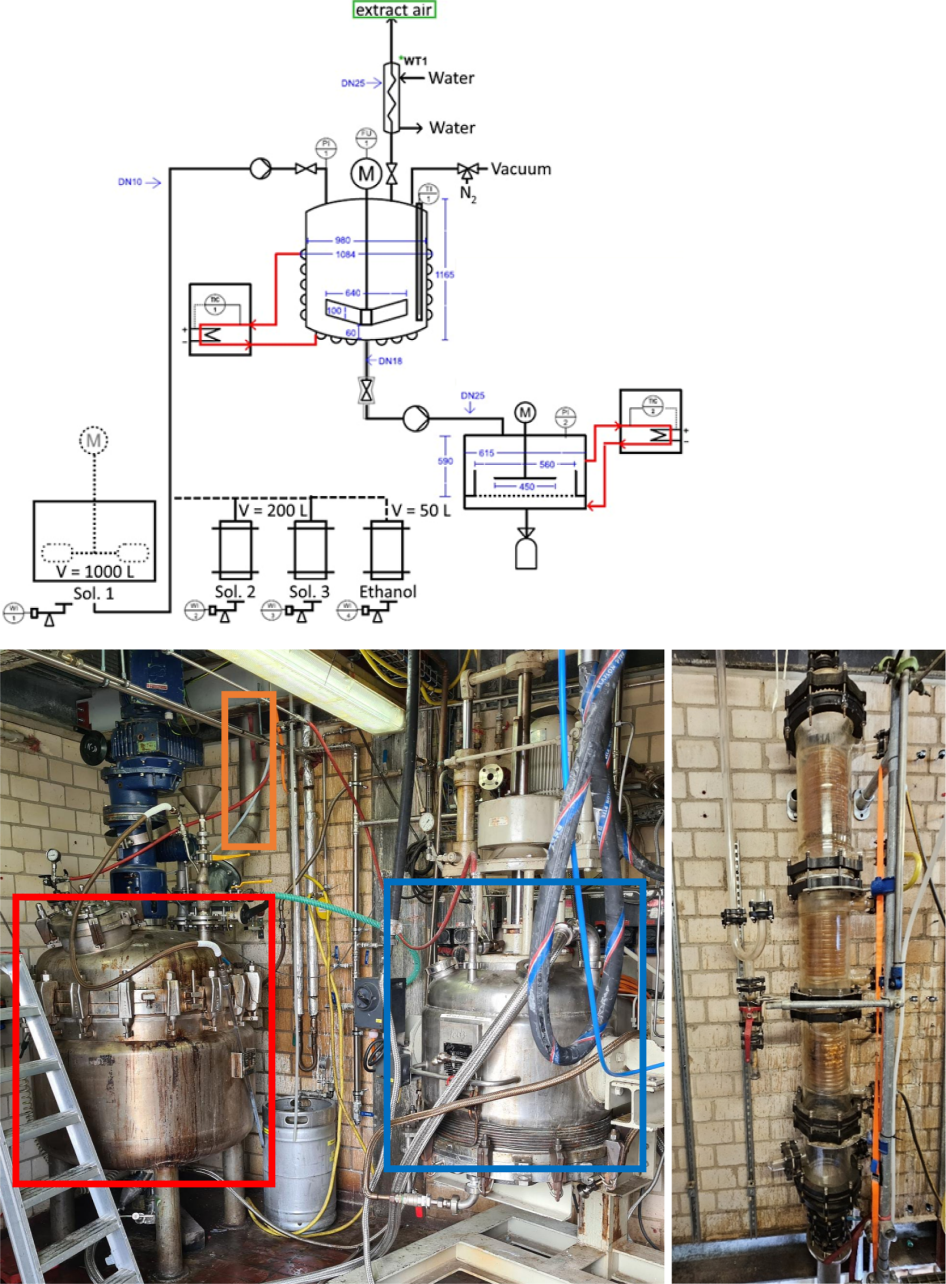
**Top**: P&ID scheme of the process setup with storage containers for the starting solutions with their respective volumes, 750 L reactor with a mechanical stirrer, a reflux condenser, and a heating element and the stirred batch filter with a heating element used for product isolation. **Bottom left**: 750 L reactor (red box), batch filter (blue box), and reflux condenser pipe (orange box). **Bottom right**: Reflux condenser used for the synthesis.

#### 3.1.2 Time Comparison of Individual Process Steps

A summary of the time required for each process step in the production of R-10 and R-750 is shown in Figure 2, and values are given in the Supporting Information in Table S6. In addition, literature data reporting the synthesis at a 50 L scale in a 200 L reactor^31^ and for an improved process are compared as well. Since the preparation of the starting solutions strongly depends on the different scales (see Supporting Information, Section S2), these times are not included in the graph. It is important to note that this diagram can only give a rough comparison between the different scale-up procedures since the reported 50 L scale synthesis lacks details about preparation and transfer of starting materials, heating, filtration, and washing time as important process steps. Details of how the times were estimated are given in the Supporting Information, Section S2, Table S6. These values were extrapolated from the respective time requirements of R-10 and R-750.^31^

**Figure 2:**
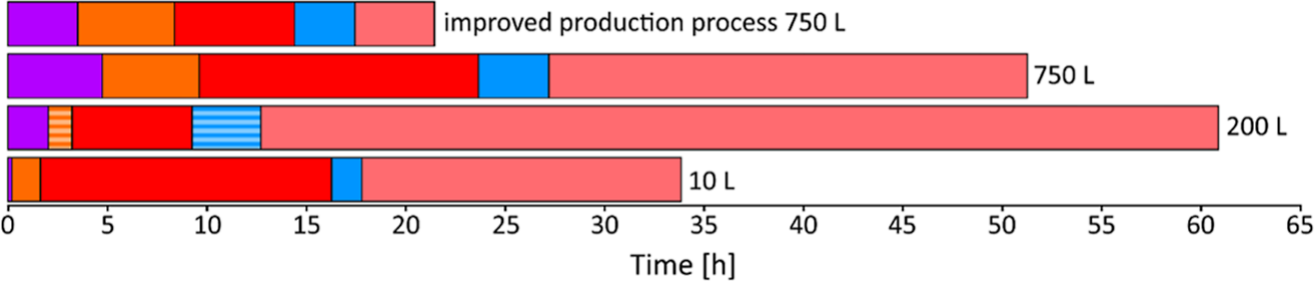
Comparison of the time necessary for each process step in the production of CAU-10-H in a 10 L glass reactor with a oil heating jacket (10 L), a 200 L glass reactor with a heating jacket (200 L),^31^ a 750 L stainless-steel reactor with a steam heating jacket (750 L), and a suggested improved production process in a 750 L reactor (750 L). **Purple** = transfer and homogenization of solutions, **orange** = heating, **red** = synthesis, **blue** = filtration and washing, and **pink** = drying. The striped areas indicate an estimated time, based on the parameters described in the literature and employing heuristic calculations to estimate the time necessary. The exact values for the different processes and details of how the times were estimated are given in the Supporting Information Section S2 and Table S6.

The total process times differ significantly between 34 and 61.5 h, and as expected, some steps scale directly with the reaction volume, *i*.*e*., transfer and homogenization of starting solutions (purple) and heating time (orange). The reaction temperature is reached within 1 h 25 min and 4 h 50 min, respectively. The time for heating of the reaction mixture to the desired reaction temperature strongly depends on the available equipment, and in the case of R-750, several steps with different heating rates had to be applied to prevent rapid expansions within the heating rack, which otherwise could potentially damage the system. For R-10, a constant heating rate of ∼60 °C h^–1^ could be applied. The reaction times (red) vary considerably, but this is due only to organizational aspects. Time-resolved synthesis studies have shown that a reaction time of 6 h is sufficient for full crystallization. Filtration and washing (blue) proved to be much faster for R-750 in relation to the treated volume. This was due to the use of a stirred, heated, and pressurized batch filter (120 °C, 2–2.5 bar excess pressure), which allowed rapid isolation and hot washing of the product. The time necessary for drying of the reaction product (pink) is not optimized, and although very long, it is usually not the time-determining step since it can be carried out in parallel to the next synthesis batch in a separate piece of equipment.

The following steps should be considered in setting up an improved process. The transfer of the starting solutions, which was carried out in R-750 by using negative pressure, can only be accelerated by using higher vacuum, active pumps, or elevated storage containers of the starting solutions for transfer by gravity. Homogenization of the starting solutions can be shortened significantly by employing higher stirring rates and controlling the viscosity of the reaction mixture to determine the optimal point of addition of the next starting solution. Reduction of the heating time can be accomplished by using the highest heating rate possible. Improvements in reaction times are not possible since a minimum time of 6 h for full crystallization is necessary. It could be shortened only by using pressurized equipment and higher reaction temperatures.

Taking that information on possible time optimizations into account, an improved production process at a 750 L scale using the available equipment could be finished within 21 h 20 min (Figure 2, Table S6) by shortening the transfer and homogenization of solutions by *ca*. 1 to 3 h 30 min (purple). Heating to the reaction temperature will be the same (4 h 50 min) since the same device would be used. The reaction time (red) can be reduced to 6 h, and the filtration and washing phase can be reduced to 3 h by carrying out one washing step with a larger volume. The drying stage of the products has great potential for time savings and can be shortened to around 4 h by using a rotary dryer, consequently increasing the STY substantially.

#### 3.1.3 Product Properties

The reaction products R-10 and R-750 were characterized by PXRD, N_2_ and H_2_O sorption measurements, TGA, EA, SEM/EDX, SLS, and MIR and Raman spectroscopy (Supporting Information, Section S3). PXRD data (Figure 3 **left**) confirms the phase purity and high crystallinity of the reaction product and the formation of the hydrated phase (CAU-10-H_aq_). The volumetric N_2_ sorption isotherms at 77 K (Figure S6) are almost identical and resemble the expected type I isotherm for microporous compounds. The apparent specific BET surface area of A_BET_ = 679 m^2^ g^–1^ and the micropore volume V_mic_ = 0.263 cm^3^ g^–1^ for R-750 are slightly higher but nevertheless in good agreement with the ones reported in the literature.^23,38–40^ The gravimetric H_2_O sorption isotherms of R-750 and R-10 at 298 K are shown in Figure 3 **right**. The characteristic S-shape is observed with the adsorption starting at ∼17% RH and at 30% RH, around 308 mg g^–1^ water is adsorbed, corresponding to 84% of the total adsorption capacity at maximum relative humidity. The desorption branch shows no obvious sign of a hysteresis loop, indicating a continuous water sorption process as expected for high-quality CAU-10-H. The characterization data demonstrate that phase-pure CAU-10-H can be synthesized in large amounts by linear scale-up of the existing green synthesis protocol optimized for a 10 L batch reactor.

**Figure 3:**
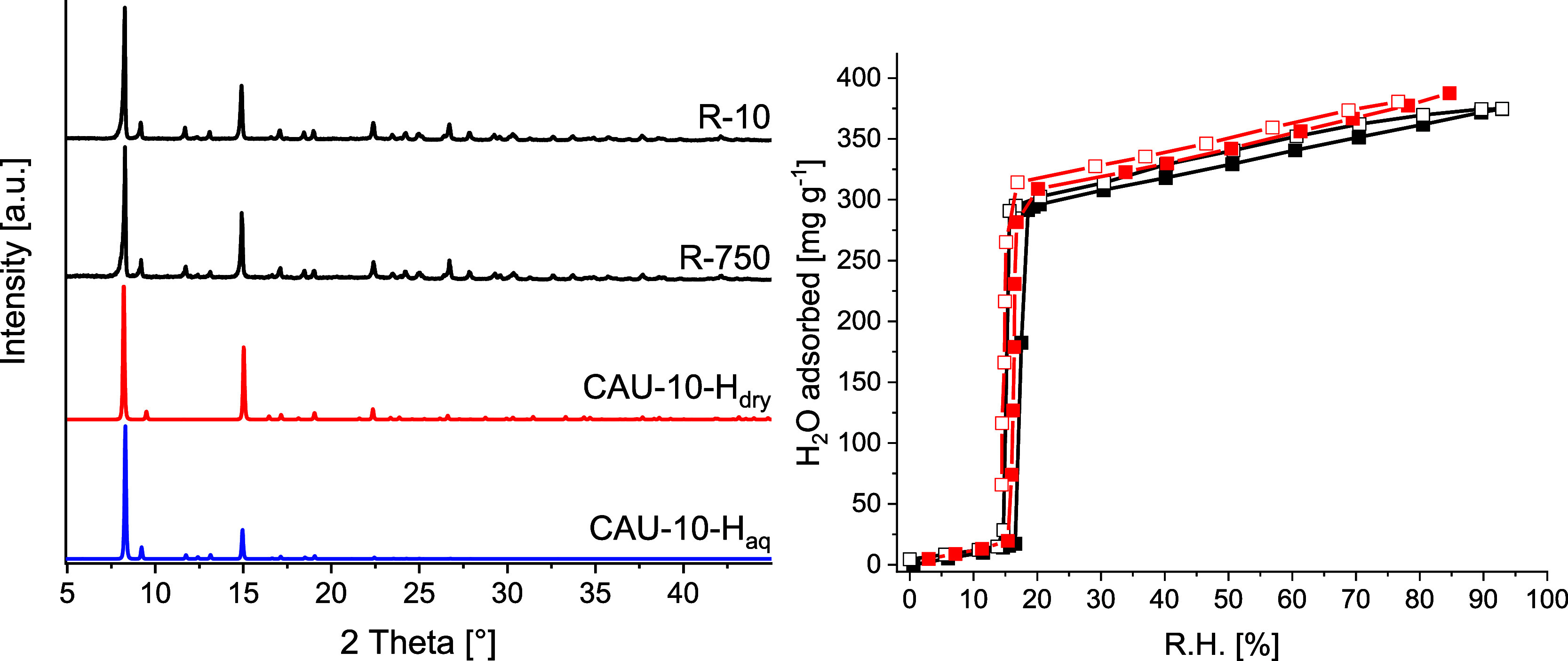
**Left**: PXRD data for R-10 and R-750 together with simulated PXRD data of wet and dry CAU-10-H, *i*.*e*., CAU-10-H_dry_ (CCDC 1454067) and CAU-10-H_aq_ (CCDC 1454066). **Right**: H_2_O sorption isotherms of R-10 (red) and R-750 (black) recorded at 298 K. Filled symbols indicate the adsorption branch, empty symbols indicate the desorption branch.

### 3.2 Techno-Economic Analysis

In this section, we propose an industrial-scale batch process production based on the synthesis at a pilot scale (750 L scale) with a reactor STY of *ca*. 99 kg m^–3^ d^–1^. The cost calculation main assumptions and methodology follow the previous studies published by some of us on Al-MIL-160, Al-MIL-120, and Fe-MIL-100.^16–18^ Further details are described in Section S4 of the Supporting Information. The rationale of the industrial process, represented as a flowsheet in Figure S12 in the Supporting Information, followed the same methodology as the scaled-up synthesis protocol where the raw materials are prepared in solutions 1, 2, and 3 and then fed to a batch stirred reactor, followed by filtration to collect the resulting solid, washing with boiling water, drying, and storage.^16^ The major equipment considered for the techno-economic analysis (TEA) includes storage bins/tanks for each reactant, the final product, ethanol, and solutions 1, 2, and 3. A propeller agitator is considered for the agitation of the tanks for solutions 1, 2, and 3. The synthesis of CAU-10-H is considered to be carried out in a batch jacketed and stirred reactor with a turbine agitator. The process also includes a plate-and-frame filter to filter and wash the solid material produced in the reactor, a rotary dryer to dry the washed solid, and a conveyor belt to the transport dried CAU-10-H to a storage bin. All the equipment size and operations were designed for a yearly production of 1 kt, as a first approach, to provide a more accurate comparison with the literature aforementioned.^16–18^ The design and dimensioning of the process equipment took into consideration common chemical engineering heuristics, material properties, process conditions, the production target (1 kt y^–1^), considering a first scenario with a reaction yield of 95% and an STY of *ca*. 99 kg m^–3^ d^–1^ (based on the extrapolated values of R-750, as described earlier) and a second scenario with a reaction yield of 98.9% and an STY of 481 kg m^–3^ d^–1^ (based on the synthesis conditions of R-750i and the reagents concentration of R-10-IC1, 1 mol L^–1^), and a yearly operation of 260 days, following the approach used by some of us in another work.^18^ The TEA includes the estimation of the total investment required for an industrial-scale CAU-10-H production plant and the estimation of the production cost of this MOF. A detailed description of the methodology and assumptions used can be found in Section S4 of the Supporting Information.

The total investment for the first scenario (STY of *ca*. 99 kg m^–3^ d^–1^) was estimated to be *ca*. 15.2 M$ (2022 prices), which includes an investment in base equipment of *ca*. 2.9 M$ (2022 prices) and a working capital of *ca*. 2.4 M$ (2022 prices), as detailed in Tables S8 and S9 in the Supporting Information.

The production cost for the yearly production of 1 kt of CAU-10-H was estimated to be *ca*. 13.8 ± 2.8 M$ (2022 prices), from which the raw materials (listed in Table S10 in the Supporting Information) account for *ca*. 3.9 M$ (2022 prices), as detailed in the Supporting Information and in Table S11. This production cost estimated for 2022 was scaled to 2019 for a more accurate comparison with previous estimates for other materials. The production cost scaled down to 2019, 10.4 ± 2.1 $ kg^–1^, is much lower than the estimate for Al-MIL-160 (29.5 $ kg^–1^ for 1 kt y^–1^),^16^ which is mainly due to the less expensive starting raw materials used for CAU-10-H. In fact, since Al-MIL-160 is based on 2,5-furandicarboxylic acid (FDCA), which is currently being considered for bioplastics production, it may have an advantage in the future.^41^ Yet, it is still dependent on the decrease of the market price of this ligand to reach cost values around 10 $ kg^–1^, closer to the production cost estimated herein for CAU-10-H. Comparing with Fe-MIL-100, another benchmark MOF, with iron as the metal source and trimesic acid as the ligand, the CAU-10-H production costs are also lower than those estimated for Fe-MIL-100 (30 $ kg^–1^ for 1 kt y^–1^).^17^ The estimated production cost of CAU-10-H is in the same range as that recently estimated for Al-MIL-120 (10–13 $ kg^–1^ for 1 kt y^–1^).^18^ The calculated cost of CAU-10-H is on the lower end of costs projected/reported in the literature for MOFs (<14 to 33 $ kg^–1^) but estimated by much less accurate economic analysis that do not consider the investment and operation costs.^17^ For the second scenario, considering an optimized CAU-10-H production process with an increased STY of *ca*. 481 kg m^–3^ d^–1^ (further details given in Section S5 in the Supporting Information), there is a decrease in the production costs to *ca*. 12.1 ± 2.4 $ kg^–1^ (2022 prices, additional details in Tables S12 to S14 in the Supporting Information). This reduction is mainly due to a reduction in the capital costs of some equipment prices, namely, the smaller reactor and tanks of solutions 1, 2, and 3 (and respective agitators), the use of a lower amount of water and ethanol as solvents (due to the increase of the concentration of the starting solutions from 0.5 to 1 mol L^–1^), and a reduction in the energy consumption (less volume to process and drying time to achieve the same yearly production). Nevertheless, despite an almost 5-fold increase in the STY (*i*.*e*., from 99 to 481 kg m^–3^ d^–1^), the overall cost reduction amounted only to 13%. This is mainly due to the fact that some costs were maintained in the remaining production process (namely, reagents (with only minor differences due to an improved synthesis yield), filtering, transport, and CAU-10-H storage). For a visual comparison of the cost structure of the direct costs of the manufacturing costs for the respective STYs, see Figure S13 in the Supporting Information.

Finally, a sensitivity analysis was performed to have a better comprehension of the influence of some of the main inputs of the economic model on the final production cost. The first selected inputs were the ligand and electricity prices as they are considered to be more susceptible to variations, especially the electricity price, which has increased substantially in the past few years.^42^ The remaining raw materials (sodium aluminate, sodium hydroxide, aluminum sulfate octadecahydrate, and ethanol) are common raw materials used in the industry, and their price variation was not considered in this sensitivity analysis. Additionally, we have performed a sensitivity analysis on the cost of the base equipment, considering that the cost of chemical engineering equipment has increased considerably in recent years^43^ and that estimates of investment based on historical equipment prices are approximations, as well as on the cost of operating labor, which can vary significantly for different countries. The results of the sensitivity analyses for the first scenario (STY of *ca*. 99 kg m^–3^ d^–1^) are displayed in Figure 4. The ligand price (Figure 4 **left**) has a more pronounced influence on the production cost, which varies from 12.8 to 16.4 $ kg^–1^ when the price of the ligand fluctuates between 1.1 and *ca*. 3.7 $ kg^–1^. More specifically, the MOF cost will increase by *ca*. 18% if the linker price doubles (a 100% increase compared to the current price considered, *cf*. Table S10 in the Supporting Information). In Figure 4 **left**, we can also see that the variation of the energy price has a lower influence on the final production cost, which ranges from 13.0 to 14.9 $ kg^–1^ when the energy prices vary from 0.032 to 0.310 $ kWh^–1^. Notably, the cost of the MOF will increase by *ca*. 8% if the energy price doubles (a 100% increase in electricity price). Regarding the influence of the base equipment and operating labor costs (Figure 4 **right**), a variation of ±20% (a common uncertainty of the cost factor estimation method) on the cost of the base equipment fluctuates the final MOF cost by ±1.0 $ kg^–1^ (±7% on the base scenario MOF cost) that on the cost of operating labor by ±0.6 $ kg^–1^ (±4% on the base scenario MOF cost). These sensitivity analyses reveal a mild variation in the production cost of CAU-10-H. Furthermore, considering the principle of the economy of scale, the production cost could decrease even further for larger production scales. Overall, the techno-economic and complementary sensitivity analyses performed attest to a promising real-world industrial production scenario based on common industrial raw materials and green and low-cost protocols, which result in a production cost of CAU-10-H that is robust against fluctuations in ligand, energy, and base equipment prices and operating labor costs.

**Figure 4:**
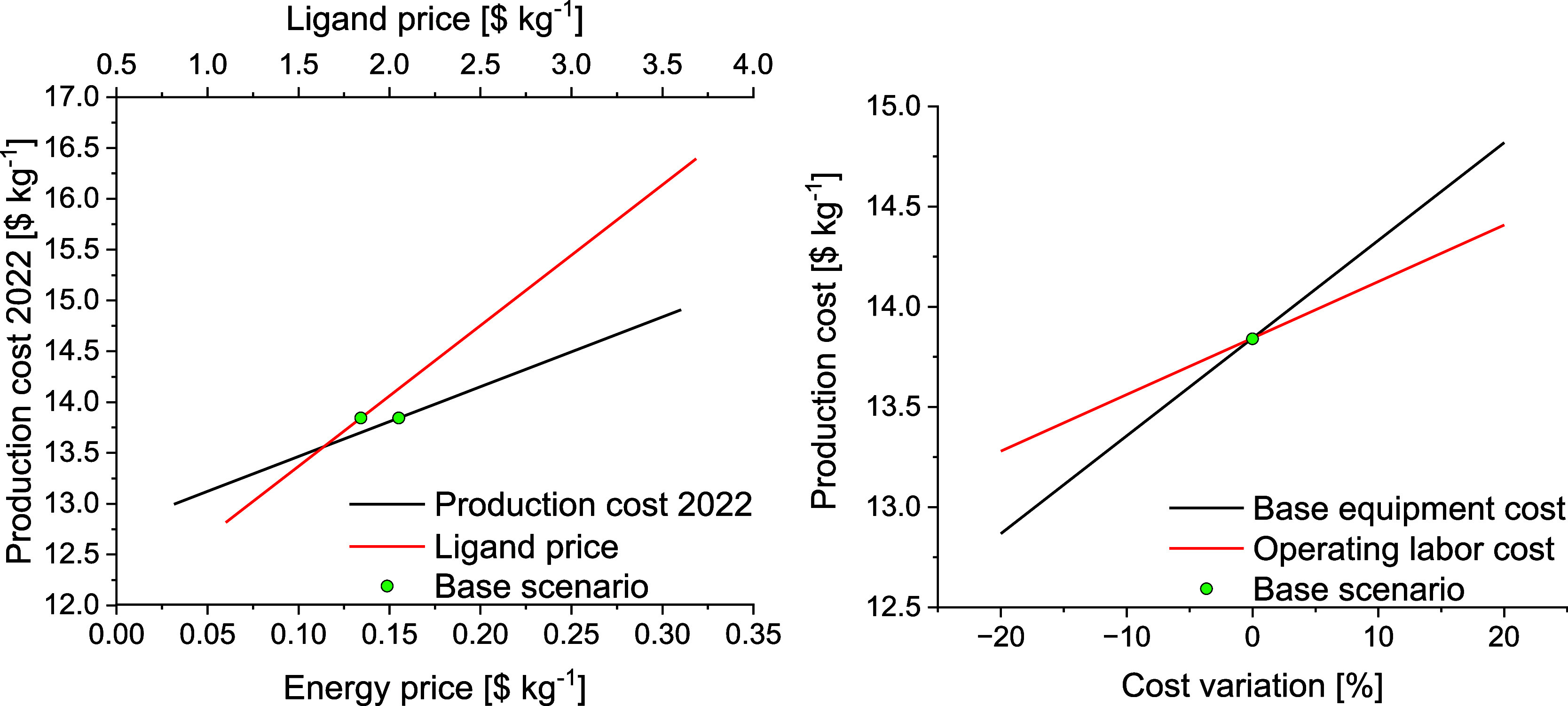
Sensitivity analysis of CAU-10-H production costs for the first scenario considered (STY of *ca*. 99 kg m^–3^ d^–1^). **Left**: Effect of the ligand (red) and energy (black) costs; **right**: effect of the base equipment costs (black) and the operating labor cost (red). The green dots over the lines represent the base economic scenario (2022 prices).

### 3.3 Further Process Optimization

In addition to time aspects more related to the equipment and process steps, as outlined in Section 3.1.1. and Figure 2, which directly lead to shorter processing times, increasing the synthesis concentration will even more strongly impact the STY. Aspects such as modifying process steps and recycling the solvent to reduce overall costs can also be important. These have been studied making use of the 10 L reactor setup. Details are given in Section S5 in the Supporting Information.

Changes in the process steps that would result in shorter transfer and homogenization times and lower energy consumption include the preparation of solution 1, *i*.*e*., dissolving sodium hydroxide and isophthalic acid in the reactor, which is exothermic. In the R-10 scale, the temperature of solution 1 reached up to 60 °C, with direct addition of ethanol and solutions 2 and 3, and starting the heating process right away would shorten this process phase.

The recycling of the solvent should be considered for a cost-effective and environmentally friendly large-scale production of CAU-10-H. Syntheses in the 10 L reactor demonstrate that recycling of the solvent twice does not compromise the yield, and only slight changes in sorption behavior of the final material are observed (Figure 5 and Supporting Information Section S5). Consequently, the cost of buying and disposal of the solvent can be reduced by a factor of 3. PXRD patterns, volumetric H_2_O sorption isotherms, and results of the gravimetric two-point H_2_O sorption experiments at 10% RH and 30% RH are shown in Figure 5 and Table 2. Full characterization of the synthesis products of the recycling experiments and synthesis with increased concentrations is given in the Supporting Information Section S5.

**Figure 5:**
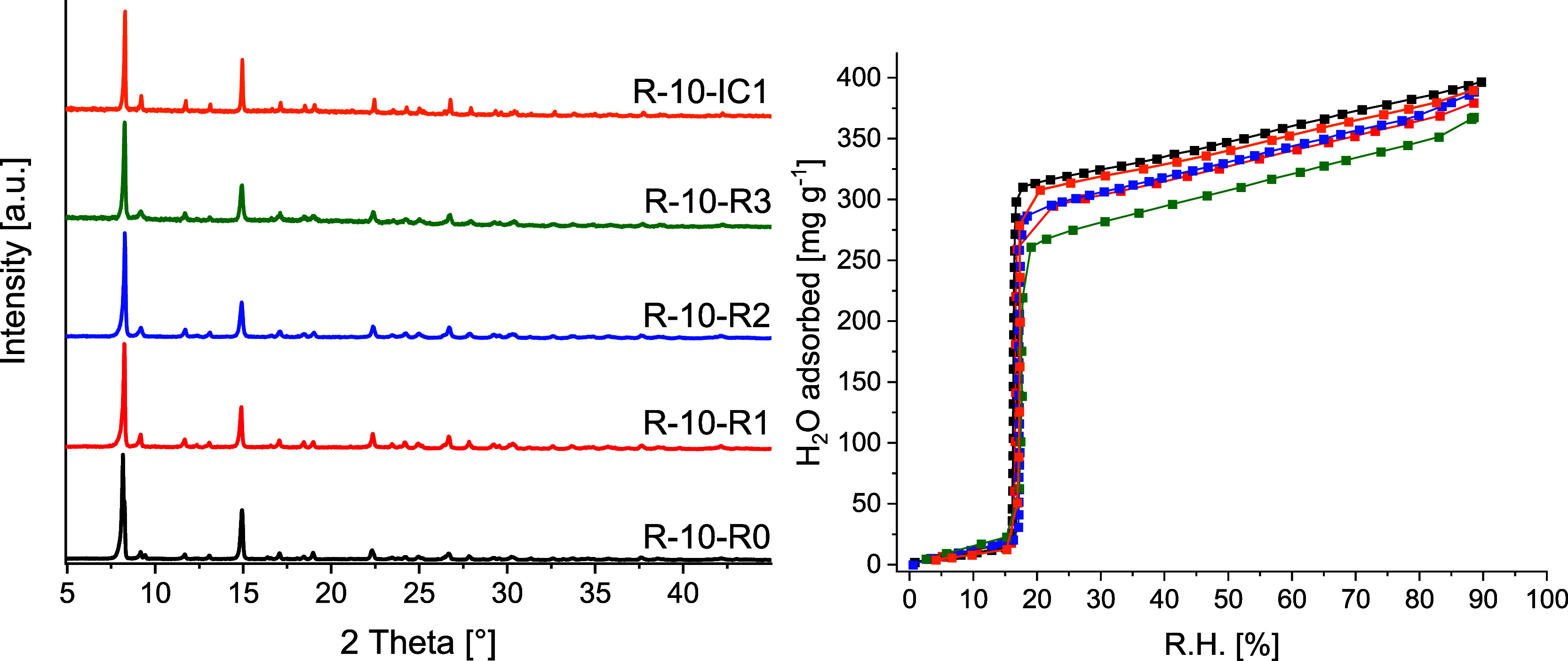
**Left**: PXRD patterns of 10 L lab-scale synthesis products from syntheses with recycled solvent (R-10-R0 for fresh solvent to R-10-R3 for three times recycled solvent) and of the 10 L lab-scale synthesis product from synthesis with an increased concentration of 1 mol L^–1^ (R-10-IC1). **Right**: H_2_O sorption isotherms of 10 L lab-scale synthesis products from syntheses with recycled solvent R-10-R0 (black), R-10-R1 (red), R-10-R2 (blue), R-10-R3 (green), and R-10-IC1 (orange) at 298 K. Desorption branches are omitted for clarity.

**Table 2:** Yields and H_2_O Uptake at 10% RH and 30% RH of Products from Syntheses with Recycled Solvent (R-10-R0 to R-10-R3) and Increased Concentration (R-10-IC1)

product from synthesis with	yield_linker_ [%]	H_2_O uptake_10% RH_ [mg g^–1^]	H_2_O uptake_30% RH_ [mg g^–1^]
R-10-R0	92.3	7	324.6
R-10-R1	91.7	8	303.4
R-10-R2	92.9	9	305.7
R-10-R3	95.8	17	280.8
R-10-IC1	98.9	8	318.7

The STY can also be increased by increasing the synthesis concentration. Therefore, additional experiments have been conducted doubling the concentration of the starting materials, *i*.*e*., using concentrations of 1 mol L^–1^ for the aluminum salt and linker solutions (Table S5). PXRD data and the H_2_O sorption isotherms at 298 K are presented in Figure 5 and confirm the successful synthesis of CAU-10-H without compromising its properties. Full details on the synthesis and characterization are given in the Supporting Information Section S5.

The results proved the successful synthesis of high-quality CAU-10-H under these conditions. An improved synthesis process, as visualized in Figure 2, using the available equipment leading to a STY of 481 kg m^–3^ d^–1^ taking the results of this section into account is given in the Supporting Information S5.

### 3.4 Evaluation of CAU-10-H for ADC

For the application of CAU-10-H in ADC devices, the adsorbent material must be mechanically and thermally fixed on an adsorber heat exchanger. The quality and thickness of the adsorbent layer are important factors for stability and performance.

Coating samples were prepared on 50 × 50 × 1 mm aluminum plates; see Section S6 in the Supporting Information for a description of the coating process. The prepared samples are denoted as C1 and C2. The adsorption rate of the samples was measured using a custom-built setup described elsewhere.^44^ A description and the P&ID scheme of the measurement setup are presented in the Supporting Information Section S6 and Figure S16. The measured adsorption rates for the CAU-10-H samples (normalized to the surface area, Figure 6 left) are similar to those for samples for the established adsorbents silica gel and SAPO-34, indicating the competitiveness of CAU-10-H for technical applications. Related to the dry adsorbent mass (Figure 6 **right**), CAU-10-H shows even more rapid adsorption than the established adsorbents.

**Figure 6:**
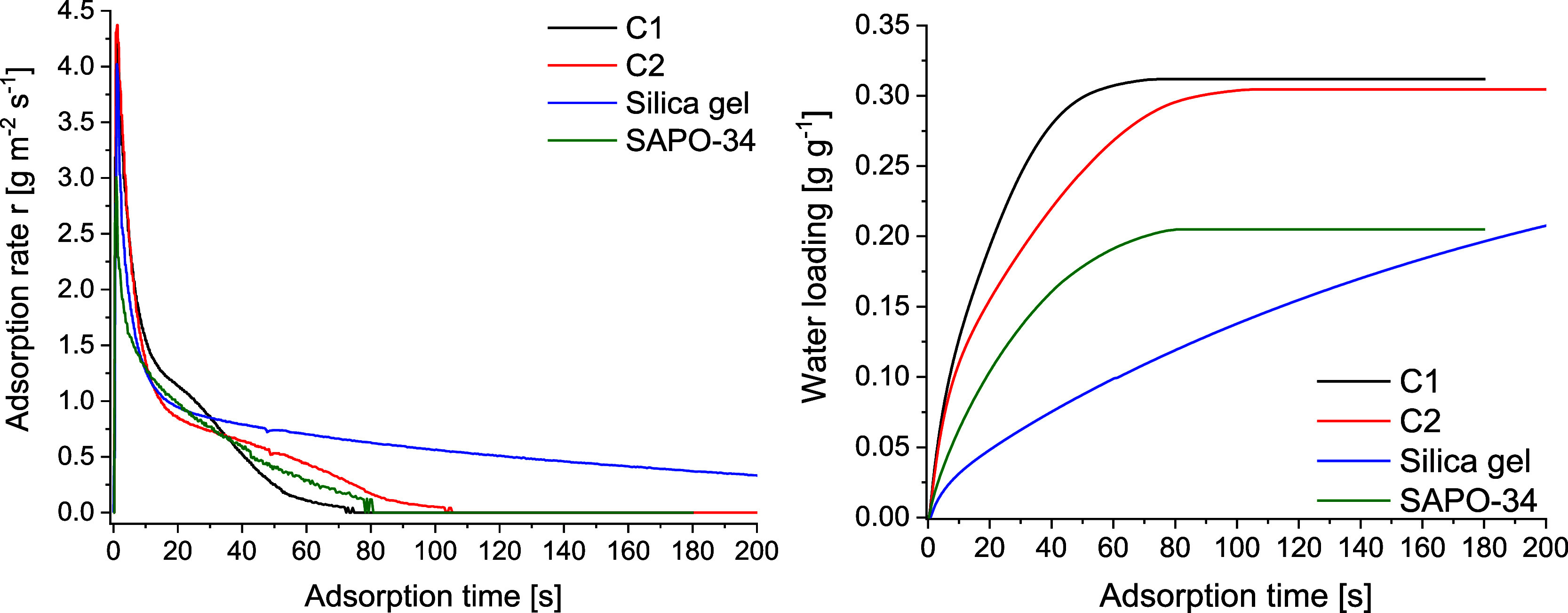
**Left**: Comparison of the specific water uptake rates (gram of water vapor per square meter per second) for two CAU-10-H coatings with active masses of 195 g m^–2^ (C1; black) and 226 g m^–2^ (C2; red). The uptake rates of a silica gel sample with an active mass of 633 g m^–2^ (blue) and a SAPO-34 sample with an active mass of 270 g m^–2^ (green) are shown for comparison. **Right**: Integrated water loading of the dry adsorbent. Adsorption conditions: 30 °C (sample temperature) and 30 mbar (vapor pressure).

Kinetic and equilibrium measurements (conducted in the same setup on the same samples) were used to parametrize a dynamic model of the CAU-10-H layer in Dymola/Modelica using the open-source library SorpLib, as described elsewhere for SAPO-34 samples.^44^ A detailed Dymola/Modelica model of the adsorption chiller, which is used at SorCool for the development of silica gel and SAPO-34 chillers, was modified with the CAU-10-H model to create a simulation model of a CAU-10-H adsorption chiller. With this, simulations were conducted for different application scenarios and compared to silica gel and SAPO-34 chillers (Table 3). Based on this data, the performance of an ADC unit containing a CAU-10-H coated heat exchanger can be projected. Except for the data center and electronics cooling (low HT), the CAU-10-H chiller shows the highest thermal efficiencies (COP). This implicates less heat rejection for the same cooling power via a recooling unit, allowing the latter to be smaller and, thus, less expensive. For BAFA and both Air conditioning Europe standards, CAU-10-H is economically advantageous compared to SAPO-34, which provides more cooling power but at a smaller COP. For air conditioning on ships and ferries as well as process cooling with moderate HT heat, CAU-10-H shows both higher COP and cooling power than the other chillers. According to the TEA given in Section 3.2, CAU-10-H can be produced more cost-effectively than SAPO-34. The combination of similar or even higher cooling power with higher thermal efficiency (less expensive recooling) makes a CAU-10-H chiller a valuable addition to existing technologies. Indeed, since CAU-10-H is more efficient, a smaller adsorbent amount is used, and the equipment size would also be smaller for the same cooling power. Only considering the adsorbent amount, the investment would be at least 24 $ lower for a 20 kW cooling power adsorption chiller (considering a silica gel price of 5 $ kg^–1^; the turnover value is 4.56 $ kg^–1^). Although the economic savings in material investment are marginal, considering the energy efficiency, the cost savings are more significant, of about 673 $ y^–1^ (considering the energy tariff used for 2022, 0.155 $ kWh^–1^, and a cooling power of 20 kW).

**Table 3:** Simulated Results for Silica Gel, SAPO-34, and CAU-10-H Chillers Containing 2 Adsorber Heat Exchangers, Each of 77 Liter Size^a^,^b^

	inlet temperatures (HT/MT/LT) [°C]	silica gel chiller			SAPO-34 chiller			CAU-10-H chiller		
application		COP	P_LT_ [kW]	EER	COP	P_LT_ [kW]	EER	COP	P_LT_ [kW]	EER
BAFA reference point	85/27/15	0.58	14.1	62	0.56	**23.8**	105	**0.65**	18.0	80
air conditioning Europe 1	80/35/15	0.53	6.4	28	0.50	**11.9**	53	**0.74**	10.0	44
air conditioning Europe 2	90/30/15	0.54	12.4	55	0.61	**24.0**	106	**0.73**	17.1	76
air conditioning ships/ferries	70/25/10	0.59	8.6	38	0.50	13.0	58	**0.76**	**16.9**	75
moderate heat process cooling	70/35/15	0.51	3.7	16	-	-	-	**0.72**	**9.2**	41
data centers, electronics	55/25/20	**0.70**	**11.3**	50	0.46	5.6	25	0.26	4.4	19

a COP: thermal efficiency, P_LT_: cooling power, EER: electrical efficiency, HT = high temperature (driving), MT = medium temperature (recooling), LT = low temperature (cooling).

b The highest values per application are highlighted in bold.

## 4 Conclusion

In this study, we report the green scale-up synthesis of CAU-10-H in a batch reactor of pilot-plant scale (750 L) for the first time. 27.5 kg of dry MOF were obtained, corresponding to a yield of 88% (based on the linker molecule). Taking into account the loss of about 10% of the reaction mixture during processing, this is in good agreement with the established yields of 90–95% from smaller-scale synthesis.^21^ This results in an STY of ∼100 kg m^–3^ d^–1^, which can be improved by discussed optimizations to reach up to >400 kg m^–3^ d^–1^. Based on the obtained data, a techno-economic analysis yielded production costs of 13.8 ± 2.8 $ kg^–1^ and 12.1 ± 2.4 $ kg^–1^ for an annual production of 1 kt of CAU-10-H with STYs of *ca*. 99 kg m^–3^ d^–1^ and *ca*. 481 kg m^–3^ d^–1^, respectively. Process optimization, such as adapting the synthesis protocol and recycling the solvent, can reduce this price even further. This demonstrates that the cheap, large-scale production of CAU-10-H is possible and cost-effective.

The evaluation of CAU-10-H coatings for application in ADC devices under real-operating conditions rendered higher COPs than benchmark materials SAPO-34 and silica gel for every tested condition except for data centers. Higher COPs and significantly higher cooling powers of 16.9 and 9.2 kW for air conditioning on ships and ferries and moderate heat process cooling, respectively, highlight the competitiveness of CAU-10-H to existing technology under these conditions.

This work presents the foundation for further steps toward the commercialization of CAU-10-H. For ensuring the highest efficiency in this, optimal properties of CAU-10-H and its production are to be further investigated in the future.

## Supplementary Material



## Notes

Background information on MOF commercialization, CAU-10-H properties, extended information and details for the pilot-scale synthesis of CAU-10-H, the characterization results, the techno-economic analysis, further process optimization, and the evaluation of CAU-10-H for ADC (PDF)

## Glossary

ADC: adsorption-driven cooling
AWH: atmospheric water harvesting
BE: base equipment
CAU: Christian Albrechts University
CEPCI: chemical engineering plant cost index
COP: coefficient of performance, thermal efficiency
D: direct costs
EA: elemental analysis for H, C, N, S
EDX: energy-dispersive X-ray analysis
EER: electric efficiency ratio
FI: fixed investment
I: indirect costs
*m*-H_2_BDC: 1,3-benzenedicarboxylic acid; isophthalic acid
Δ*H*_ads_: adsorption enthalpy
HT: high temperature (driving)
LT: low temperature (cooling)
MeOH: methanol
MIL: Matériaux de l′Institut Lavoisier
MIR: mid-infrared
MT: medium temperature (recooling)
MTBE: methyl *tert*-butyl ether
MOF: metal–organic framework
P_HT_: heating power
P&ID: piping and instrumentation diagram
P_LT_: cooling power
PPI: producer prices in the industry
PXRD: powder X-ray diffraction
RH: relative humidity
RP: risk provision
SEM: scanning electron microscopy
SLS: static light scattering
SS: stainless steel
STY: space-time yield
TEA: techno-economic analysis
TGA: thermogravimetric analysis
USD: United States dollar
