# Supplementary material for: CAU-10-H: Synthesis Scale-Up at the Pilot Scale, Techno-Economic Analysis, and Application in a Full-Scale Cooling System
Source: Ind Eng Chem Res. 2026 Mar 26;65(13):7045–55. doi: 10.1021/acs.iecr.5c05308 (PMC13067280; doi:10.1021/acs.iecr.5c05308)
Supplement: Supplementary file 1 [file ie5c05308_si_001.pdf]

Supplementary Information

CAU-10-H: Synthesis Scale-up at Pilot Scale, Techno-Economic Analysis and Application in a Full-Scale Cooling System

Kalle S. Mertin[a], Abeer Mohtar[b], Marta Bordonhos[b,c], Moisés L. Pinto[b,], Thomas May[d], Ralph Herrmann[d,*], and Norbert Stock[a,e,*]*

[a] Institut für Anorganische Chemie, Christian-Albrechts-Universität zu Kiel, 24118 Kiel, Germany.

[b] CERENA, Departamento de Engenharia Química, Instituto Superior Técnico, Universidade de Lisboa, Av. Rovisco Pais 1, 1049-001 Lisboa, Portugal

[c] CICECO – Aveiro Institute of Materials, Department of Chemistry, University of Aveiro, Campus Universitário de Santiago, 3810-193 Aveiro, Portugal

[d] Dr. Ralph Herrmann, SorCool GmbH, Zscherbener Landstraße 12, 06126 Halle, Germany

[e] Kiel Nano, Surface and Interface Science KiNSIS, Christian-Albrechts-Universität zu Kiel, 24118 Kiel, Germany

* Corresponding authors: stock@ac.uni-kiel.de

moises.pinto@tecnico.ulisboa.pt

ralph.herrmann@sorcool.de

Table of Contents

S1. Background on commercialization of MOFs and CAU-10-H properties 3
S2. Pilot scale synthesis of CAU-10-H 7
S3. Characterization of R-750 and comparison to R-10 12
S4. Techno-economic analysis of CAU-10-H production costs 17
S5. Further process optimization 28
S6. Evaluation of CAU-10-H for ADC 30
S7. References 32

S1. Background on commercialization of MOFs and CAU-10-H properties

Table S1 shows a list of companies offering commercially available MOF materials and/or services regarding their production. **Table S1** is reproduced with permission by the corresponding author.

Table S1: Companies and their commercially available products and/or services.[1]

Company	Products/Services	Ref.
BASF (Sigma-Aldrich as retailer)	Basolite® F300	2
	Basolite® Z377	3
	Basolite® Z1200	4
Framergy (STREM as retailer)	AYRSORB™ F100	5
	AYRSORB™ F250	6
	AYRSORB™ T125	7
	AYRSORB™ P151	8
	CAU-10(Al)	9
	HKUST-1(Cu)	10
	MIL-53(Al)	11
	MIL-101(Al)-NH_2	12
	UiO-66(Zr) and its derivatives	13–15
	ZIF-8	16
MOFapps	UiO-66(Zr) and its derivatives	17
Promethean Particles	Aluminum fumarate	18
	Fe-BTC	18
	HKUST-1	18
	MIL-53(Al)	18
	MIL-100(Fe)	18
	MOF-74(Ni)/CPO-27(Ni)	18
	MOF-74(Zn)/CPO-27(Zn)	18
	ZIF-8	18
	ZIF-67	18
ProfMOF	UiO-66(Zr)-ADC and derivatives	19
	MOF-801/UiO-66(Zr)-FA	19
	UiO-67(Zr)-BPDC	19
	UiO-67(Zr)-BPY	19
	MOF-808(Zr)	19
	CAU-10(Al)	19
Immaterial	Monolithic MOF-based solution for clean air applications	20
Mosaic Materials (acquired by Baker Hughes)	Amine-grafted MOF-based direct air capture technology unit (MOF-274)	21
novoMOF	Synthesis and scale-up of MOFs	22
Nuada	MOF-based point-source CO_2 capture filtration machines	23
Numat	ION-X: MOF-based sub-atmospheric gas storage and delivery system	24

	SENTINEL: MOF-based filter material toward chemical warfare agents (CWAs)	25
Svante	MOF (CALF-20) based carbon capture filters	26
SQUAIRTECH	ST-Sorb13: Aluminum-based MOF for formaldehyde capture	27
	ST-Cata26: Iron-based MOF for air purifying	28
Vector BioScience Cambridge	MOF-based drug delivery solution	29

Table S2: Prices for selected MOFs in the largest available quantity, based on the prices indicated on the website and a quote from nanoshell-UK Ltd.

MOF	Price	Ref.
Al-MIL-53	3 900.00 € kg^{-1}	30
Fe-MIL-53	2 850.00 € kg^{-1}	30
Cr-MIL-53	3 900.00 € kg^{-1}	30
Cr-MIL-101	4 400.00 € kg^{-1}	30
Zr-MOF-801-P	6 500.00 € kg^{-1}	30
Cu-MOF-199	5 650.00 € kg^{-1}	30
Zr-UiO-66-NH_2	7 800.00 € kg^{-1}	30
MIL-101(Al)-NH_2	297.00 € per 2 g	31
MIL-53(Al)	411.00 € per 2 g	11
Basolite® F300	1 280.00 € per 100 g	2
ZIF-8	5 530.00 € per 500 g	4

A summary of the most important storage and separation studies using CAU-10-H is given in **Table S3**. The studies highlight interesting properties for CAU-10-H, focused on carbon dioxide, hydrogen and low weight hydrocarbon separation, acetylene storage and capture of SO_2, showing the potential of CAU-10-H for a wide range of applications. One study tested a membrane of CAU-10-H, that demonstrated exceptional performance for the separation of CO_2 from CH_4 and N_2 for natural gas purification, with an unprecedented permeability of 500 Barrer and selectivity values of 42 and 95 for CO_2/N_2 and CO_2/CH_4, respectively.[32]
Another study showed the potential application in bio-ethanol steam reforming processes by producing a membrane of CAU-10-H for the separation of H_2 from CO_2 and H_2O under hydrothermal conditions. Good selectivity values of 8.9 for H_2/CO_2 and 3.6 for H_2/H_2O at 300 °C from a ternary gas mixture and a stability of up to 5 bar were obtained.[33] A CAU-10-H membrane on γ-Al_2O_3 employed for the separation of an azeotropic mixture of methanol and methyl *tert*-butyl ether (MTBE) outperformed most of the benchmark materials with a separation factor of 2041, a permeation flux of 1.76 kg m^{-2} h^{-1} and at least 7 days of continuous operation without any change in performance. Although ZIF-67 is showing better performance, taking its higher production costs due to a more expensive linker molecule (2-methylimidazole) and cobalt salts as metal source and taking its inherent environmental hazard into account, CAU-10-H stands out as the more promising material for this kind of application.

Table S3: Summary of CAU-10-H performance for separation applications.

Application	Gas	Performance			Ref.
Natural gas purification	CO_2/CH_4<br>CO_2/N_2	Selectivity<br>95 CO_2/CH_4<br>42 CO_2/N_2	Permeability<br>500 Barrer	Temp.<br>30 °C	34
Additional remarks	Membrane meets criteria for industrial application<br>Highest permeability of pure MOF-Membranes				
Bio-ethanol steam reforming	$H_2/CO_2/H_2O$	Selectivity<br>3.6 H_2/H_2O<br>8.9 H_2/CO_2		Temp.<br>300 °C	35
Additional remarks	Activated permeation mechanism<br>Good H_2 separation performance in presence of steam at 300 °C				
Liquid separation	MeOH/MTBE	Selectivity<br>2041	Permeation<br>1.76 kg m^{-2} h^{-1}	Temp.<br>50 °C	36
Additional remarks	Separation of azeotropic liquid<br>Stability at least 7 days of operation<br>Better than most common membranes for this application				
Flue gas desulfurization	SO_2/CO_2	IAST selectivity<br>29 / molar ratio: 0.01<br>27 / molar ratio: 0.1<br>25 / molar ratio: 0.5	Uptake<br>3.7 mmol g^{-1}<br>(@0.1 bar)	Temp.<br>20 °C	37
Additional remarks	76 % of total capacity at 0.1 bar<br>Beneficial for pressure swing adsorption				
Separation of H_2 isotopes (composite)	*p*- and *o*-H_2/D_2	Selectivity<br>1.7		Temp.<br>- 198 °C	38
Additional remarks	Composite: UTSA-16/CAU-10-H/γ-AlOOH<br>Stability at least 10 cycles<br>Efficient separation at 10% D_2				
Xylene isomer separation	*p*-/*m*-/*o*-<br>Xylene/Ethylbenzene	Selectivity<br>12.26 *p*/*m*<br>10.55 *p*/*o*<br>0.87 *m*/*o*		Temp.<br>250 °C	39
Additional remarks	No remarks				
Acetylene separation	C_2H_2/CO_2	Selectivity<br>3.4 C_2H_2/CO_2<br>3.3 $C_2H_2/CO_2/H_2O$	Uptake<br>89.9 cm^3 g^{-1}<br>(@1 bar)	Temp.<br>23 °C	40
Additional remarks	Stability at least 15 cycles<br>Exp. Q_{st} = 32.8 kJ mol^{-1}<br>Storage density = 392 g L^{-1} (@1 bar / 23 °C)<br>Density liquid Acetylene = 393 g L^{-1} (@1 bar / 23 °C)				

Table S4: Comparison production and activation of CAU-10-H, Fe-MIL-100, Al-MIL-120, Al-MIL-160 as candidates for application in ADC.

MOF	Synthesis	Purification	Activation	Ref.
CAU-10-H	Reflux (6 h, H_2O/EtOH)	boiling H_2O	Thermal (≥ 70 °C, 12 h, ambient pressure)	[41,42]
Fe-MIL-100	Solvothermal (160 °C, 12 h, H_2O)	1. H_2O<br>2. hot water (70 °C)<br>3. hot EtOH (65 °C)<br>4. hot NH_4F solution (70 °C)	Thermal (≤ 100 °C, ambient pressure)	[43]
Al-MIL-120	Solvothermal (210 °C, 24 h, H_2O)	H_2O	Thermal activation under dynamic vacuum	[44–46]
Al-MIL-160	Reflux (≥ 6 h, H_2O)	EtOH	Thermal (120 °C, 48 h, ambient pressure); 150 °C, 12 h, purging with He)	[42,47]

S2. Pilot scale synthesis of CAU-10-H

In the following, the most important data on the synthesis of CAU-10-H at different scales is compared. **Table S5** shows the molar ratios of raw materials and solvents employed in the syntheses at different scales (R-10, R-200, R-750) as well as the synthesis optimization experiments with increased concentration (R-10-IC) and recycled solvent (R-10-R0 for fresh solvent to -R3 for three times recycled solvent). The obtained mass yield was used to calculate the molar yield in percent based on the amount of *m*-H_2BDC, the STY with respect to the reaction time and the STY taking the overall production time into account. **Table S6** shows the time necessary for each process step in the production of CAU-10-H, which were used for calculating the STY.

Table S5: Molar ratios, concentration of the linker solution, mass yield of dry product, yield and STY with regards to reaction time and overall production time for the optimized synthesis of CAU-10-H in 10 L reactor (R-10) in comparison with syntheses in 200 L reactor (R-200), 750 L reactor scale (R-750), synthesis with increased concentration (R-10-IC) and syntheses with up to three times recycled solvent (R-10-R0 for fresh solvent to R-10-R3 for three times recycled solvent).

	m-H_2BDC	$Al_2(SO_4)_3 \cdot 18H_2O$	$NaAlO_2$	Water	Ethanol
Molar ratio	1	0.375	0.25	902	7.3
R-10	$C_{solution\ 1}$<br>0.5 mol L^{-1}	Dry material<br>484.9 g	$Yield_{m\text{-}H2BDC}$<br>93.2 %	$STY_{reaction}$<br>93 kg m^{-3} d^{-1}	$STY_{production}$<br>39 kg m^{-3} d^{-1}
R-200[42]	1	0.541	-	3608	-
	$C_{solution\ 1}$<br>0.769 mol L^{-1}	Dry material<br>3.81 kg	$Yield_{m\text{-}H2BDC}$<br>92 %	$STY_{reaction}$<br>305 kg m^{-3} d^{-1}	$STY_{production}$<br>30 kg m^{-3} d^{-1}
**Synthesis scale-up**					
R-750	1	0.375	0.25	902	7.3
	$C_{solution\ 1}$<br>0.5 mol L^{-1}	Dry material<br>29.6 kg	$Yield_{m\text{-}H2BDC}$<br>95 %	$STY_{reaction}$<br>99 kg m^{-3} d^{-1}	$STY_{production}$<br>24 kg m^{-3} d^{-1}
**Synthesis optimization**					
R-10-IC1	1	0.375	0.25	451	3.65
	$C_{solution\ 1}$<br>1 mol L^{-1}	Dry material<br>1 029.4 g	$Yield_{m\text{-}H2BDC}$<br>98.9 %	$STY_{reaction}$<br>198 kg m^{-3} d^{-1}	$STY_{production}$<br>83 kg m^{-3} d^{-1}
R-10-R0	1	0.375	0.25	902	7.3
	$C_{solution\ 1}$<br>0.5 mol L^{-1}	Dry material<br>480.2 g	$Yield_{m\text{-}H2BDC}$<br>92.3 %	$STY_{reaction}$<br>92 kg m^{-3} d^{-1}	$STY_{production}$<br>39 kg m^{-3} d^{-1}
R-10-R1	1	0.375	0.25	902	7.3
	$C_{solution\ 1}$<br>0.5 mol L^{-1}	Dry material<br>476.9 g	$Yield_{m\text{-}H2BDC}$<br>91.7 %	$STY_{reaction}$<br>92 kg m^{-3} d^{-1}	$STY_{production}$<br>38 kg m^{-3} d^{-1}
R-10-R2	1	0.375	0.25	902	7.3
	$C_{solution\ 1}$<br>0.5 mol L^{-1}	Dry material<br>483.4 g	$Yield_{m\text{-}H2BDC}$<br>92.9 %	$STY_{reaction}$<br>93 kg m^{-3} d^{-1}	$STY_{production}$<br>39 kg m^{-3} d^{-1}
R-10-R3	1	0.375	0.25	902	7.3
	$C_{solution\ 1}$<br>0.5 mol L^{-1}	Dry material<br>498.4 g	$Yield_{m\text{-}H2BDC}$<br>95.8 %	$STY_{reaction}$<br>96 kg m^{-3} d^{-1}	$STY_{production}$<br>40 kg m^{-3} d^{-1}

Table S6: Comparison of times necessary for each process step in the production of CAU-10-H on 10 L, 200 L, 750 L reactor scale and the improved production process on the 750 L scale, denoted as R-750i. Italic text indicates estimated times since they have not been reported in the respective literature.

Scale	Preparation of starting solutions*	Transfer & homogenization in reactor	Heating	Reaction time	Filtration and washing	Drying
**R-10**	1 h	10 min	1 h 25 min	14 h 35 min	1 h 30 min	16 h
**R-200**[42]	1 h	2 h	*1 h 10 min*	6 h	*3 h 25 min*	48 h
**R-750**	~8 h	4 h 43 min	4 h 50 min	14 h	3 h 30 min	24 h
**R-750i**	*1 h 30 min*	*3 h 30 min*	*4 h 50 min*	*6 h*	*3 h*	*4 h*

It is important to note that this should only give a rough comparison between the synthesis scales, because not all values are directly comparable due to missing information and scale. Values given in *italics* are estimated times. The time necessary for the preparation of the starting solutions as given here is not taken into consideration for the discussion and calculation of the STY based on the overall production time. This is because the values are not comparable due to several reasons. For one, they are highly dependent on the equipment used, which drastically affects the time necessary. Second, the scale-up facility for the synthesis of R-750 carried out the preparation of the starting solutions over the course of a complete working day, several days prior to the actual synthesis; therefore, the preparation for the R-750 synthesis was performed without agitation and not done as quickly as possible. Lastly, the literature on the scale-up synthesis in a 200 L reactor[42] (R-200) states only the preparation of solution 1 directly within the reactor with 1 h of agitation, while there was no report on the time necessary for complete dissolution of the metal salt for solution 2. Consequently, no time for the transfer of solution 1 into the reactor is reported as well. Therefore, the values for preparation as well as transfer and homogenization of starting solutions in **Table S6** represent both the reported values from the literature, while in reality higher. The time for heating in the R-200 synthesis was not reported as well, therefore it was estimated by using a heuristic water heating time calculator by fitting the efficiency conditions for the calculation with the experimental data from R-10 and R-750 synthesis first. Assuming a thermostat with 12 kW power output was employed in R-200 synthesis, which is a common power output for such devices in this scale, the time necessary to heat 50 L of aqueous reaction mixture from ambient to reaction temperature of 95 °C was estimated to take approximately 1 h 10 min. The time necessary for filtration and washing was not reported as well and is highly dependent on the equipment used. Since the filtration set up was not further described in the literature, a common vacuum filtration set up was assumed. The washing was reported to be done in a total of 4 washing steps, including redispersing the solid and repeated filtration in the same filtration set up. With a maximum of 1 bar pressure difference for a perfect vacuum, the estimated 3 h 25 min necessary for filtration and washing of the product are already quite an optimistic estimation.

All values for R-10 are determined experimentally by measuring the time for each process step. Except for the time of the preparation step, all values for R-750 are taken from the process protocol provided by the facility after the scale-up experiment.

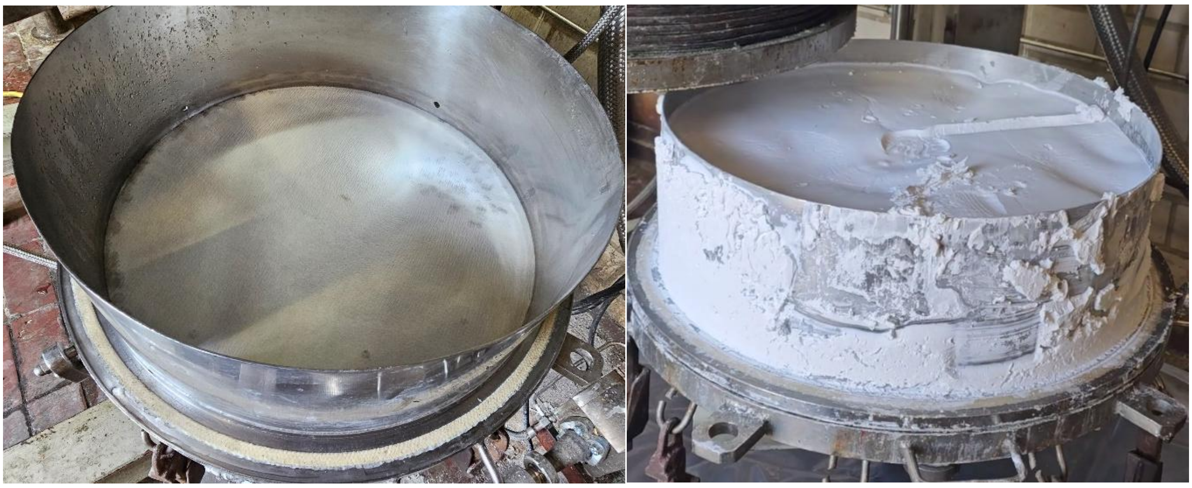

Figure S1: Bottom part of the batch filter (50 L capacity of solid) **left:** before and **right:** after product separation.

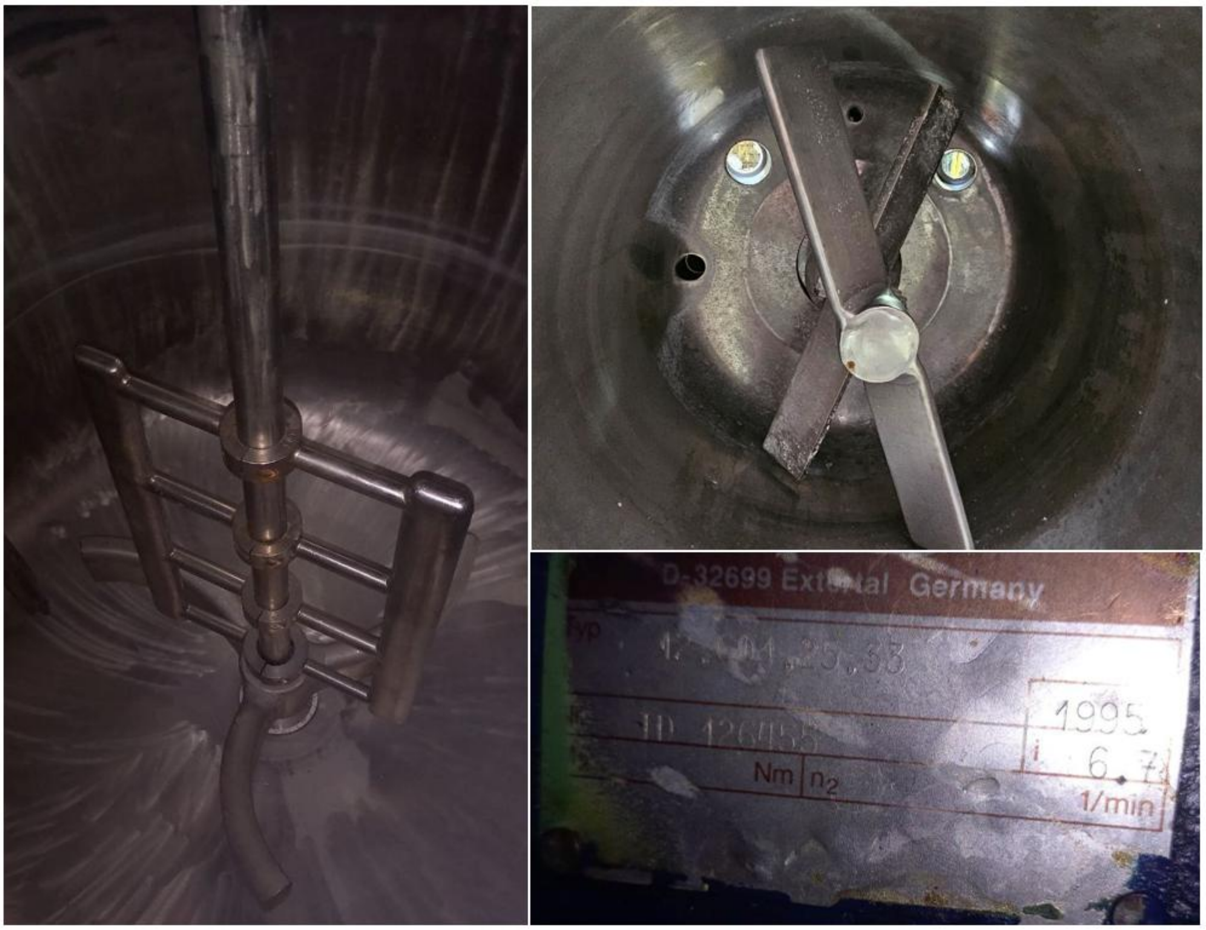

Figure S2: Agitator geometry of **left:** 750 L reactor and **top right:** nutsche funnel as well as **bottom right:** the rating plate of the agitator gearbox.

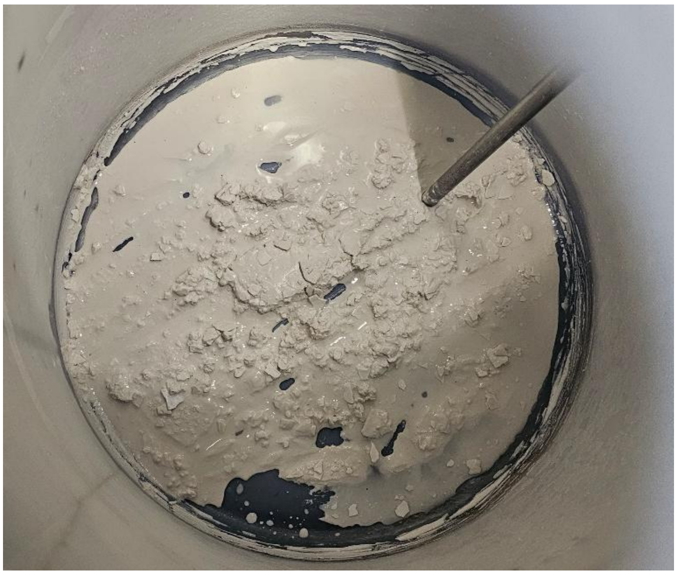

Figure S3: Residual $NaAlO_2$ in the starting solution barrel, which was dispersed with 5 L of purified water and transferred to the reactor. This is a known phenomenon happening occasionally in the established 10 L lab scale synthesis as well, with no observable impact on the final materials' properties. It appears to be dependent on the $NaAlO_2$ batch provided by the supplier.

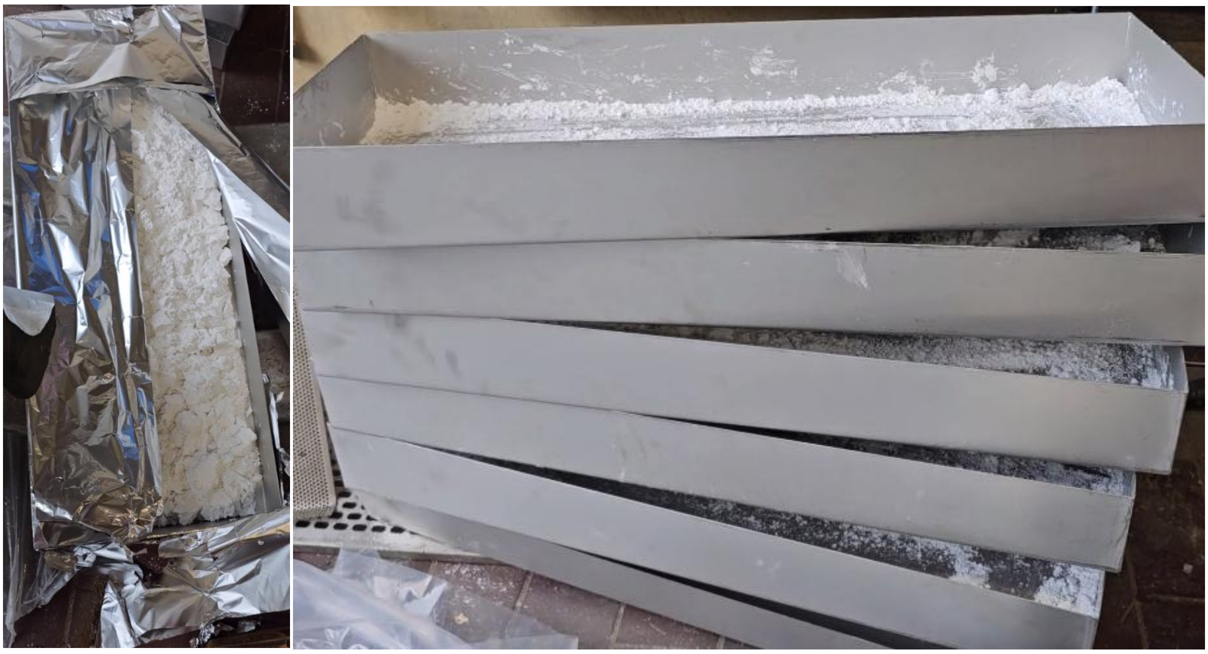

Figure S4: left: Dried product in a stainless steel container with aluminum foil cover. **right:** Emptied product containers after drying and packaging.

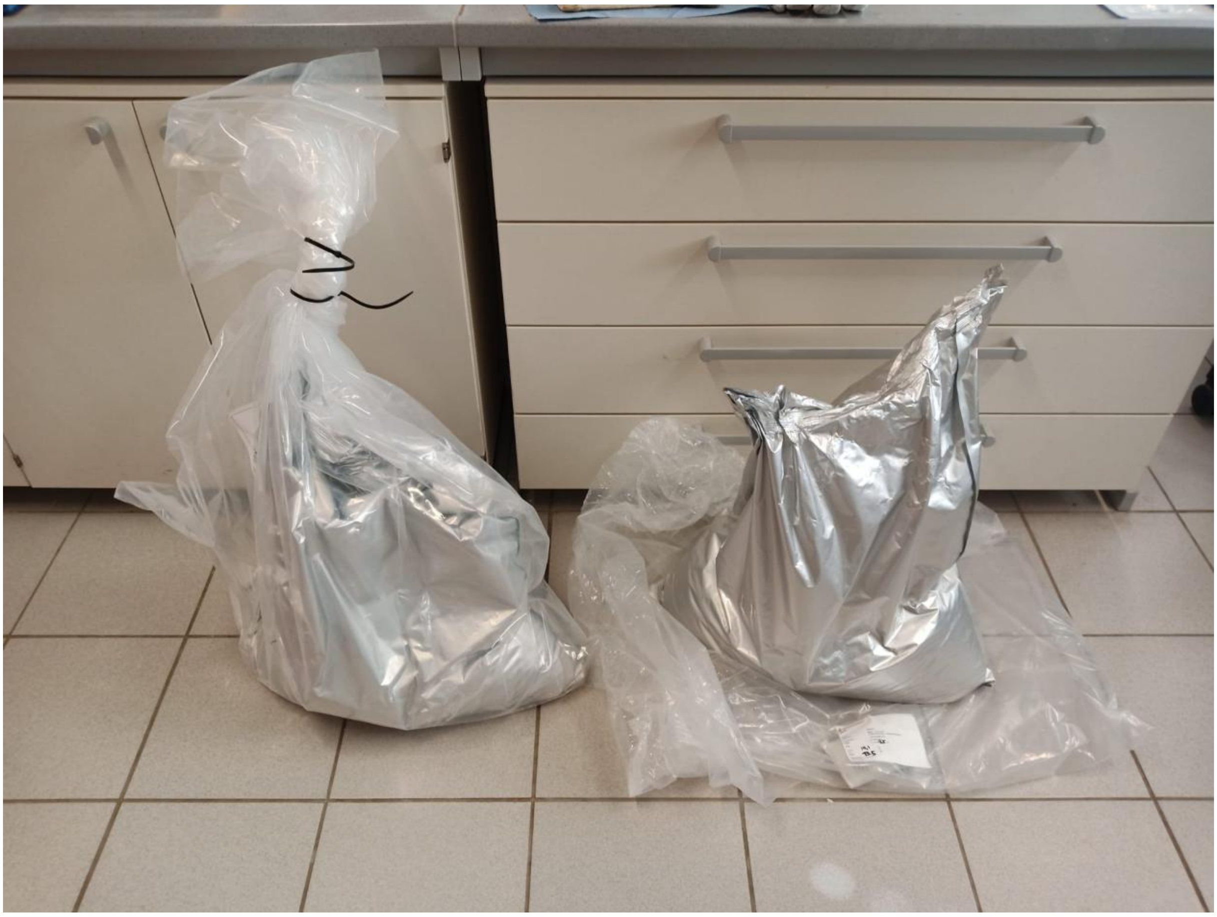

Figure S5: Aluminum bags with 27.5 kg of CAU-10-H sent to Kiel laboratories.

S3. Characterization of R-750 and comparison to R-10

In addition to the characterization data reported in the main manuscript, the results of the N_2 sorption and TG measurements, elemental analyses and scanning electron microscopy/energy dispersive X-ray analysis, static light scattering as well as MIR and Raman spectroscopy that confirm a high-quality product R-750 of the scale-up synthesis are presented.

Sorption experiments The N_2 sorption isotherms of the scale-up product R-750 (black) and the reference batch R-10 (red) are shown in **Figure S6**. BET Analysis with fitted Rouquerol criteria gave an apparent specific surface area A_{BET} = 679 m^2 g^{-1} and A_{BET} = 695 m^2 g^{-1} for R-750 and R-10, respectively.[48] The micropore volume was calculated to be V_{mic} = 0.263 cm^3 g^{-1} and V_{mic} = 0.2666 cm^3 g^{-1} for R-750 and R-10, respectively.

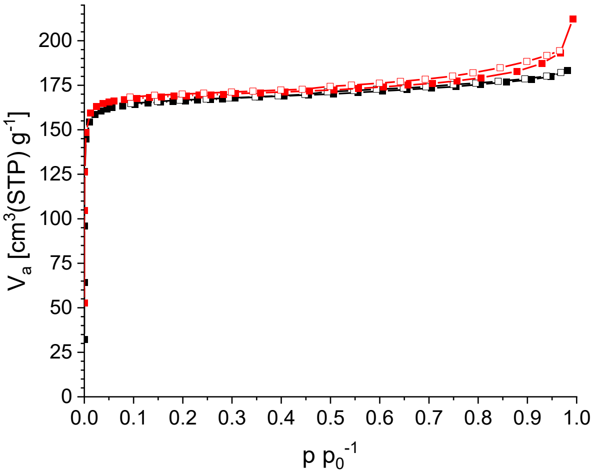

Figure S6: N_2 sorption isotherm of R-750 (black) and R-10 (red) recorded at 77 K.

TGA In **Figure S7** the TGA curves of R-750 (black) in comparison to the reference from the 10 L scale synthesis R-10 (red) are shown. The characteristic steps for phase pure CAU-10-H are clearly visible and only show small deviation from the reference, which can be explained by different amounts of adsorbed water on the sample before measurement. The first mass loss up to 150 °C is attributed to the desorption of physisorbed water inside the pores. The second step from 380 °C up to 500 °C can be attributed to the condensation of the μ_2-OH groups and is considered as the starting point of collapse of the framework. The final mass loss from 500 °C to 640 °C resembles the complete collapse of the framework, by combustion of the organic linker molecules. Based on the MIR and Raman spectra, the X-ray amorphous residue from the TGA measurement is proposed as Al_2O_3. The purity of the sample can be determined from the relative residual mass. A value of 24.49 % as relative residual mass for a pure sample of CAU-10-H is expected, taking the sample's mass at 300 °C and 640 °C. A lower value indicates residual linker molecules present in the sample, while a higher value indicates remaining Al-salts or X-ray amorphous aluminum oxide hydroxide. A value of 22.97 % was calculated for R-750, indicating a small amount of unreacted *m*-H_2BDC species in the product of the scaled-up synthesis, which is reasonable due to the complications during processing and the poor solubility of *m*-H_2BDC in water.[49,50]

Elemental Analysis The results from the elemental analysis are shown in **Table S7**. Together with the TG analysis these correspond to phase pure CAU-10-H with the sum formula $[Al(OH)(C_8H_4O_4)] \cdot 4.2\ H_2O$.

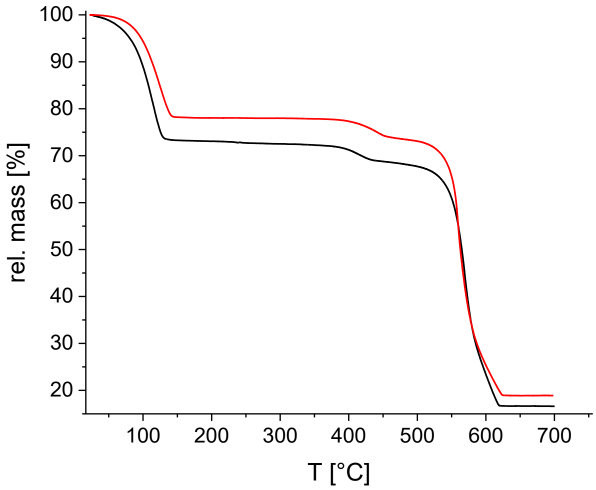

Figure S7: TG curves of R-750 (black) and R-10 (red).

Table S7: Observed and calculated mass loss steps of the TG data and observed and calculated values for the elemental analysis results of R-10 and R-750.

**Sample**	**R-10**		**R-750**	
Sum formula	$[Al(OH)(C_8H_4O_4)] \cdot 3.25\ H_2O$		$[Al(OH)(C_8H_4O_4)] \cdot 4.2\ H_2O$	
**TGA**	Obs.	Calc.	Obs.	Calc.
Solvent step [%] T = 20 – 300 °C	22.01	21.96	27.49	26.66
Framework step [%] T = 300 – 700 °C	59.09	58.92	55.87	55.37
Relative residual mass [%]	24.23	24.49	22.97	24.49
**Elemental Analysis**				
C ± 0.06 [%]	36.76	36.16	33.86	33.86
H ± 0.05 [%]	4.70	4.32	4.43	4.76
N ± 0.14 [%]	0	0	0	0
S ± 0.08 [%]	0	0	0	0

SEM/EDX Analysis R-750 was studied by means of SEM/EDX analysis. **Figure S8** shows a SEM micrograph of R-750 and the measured EDX spectrum (marked spot in SEM picture).

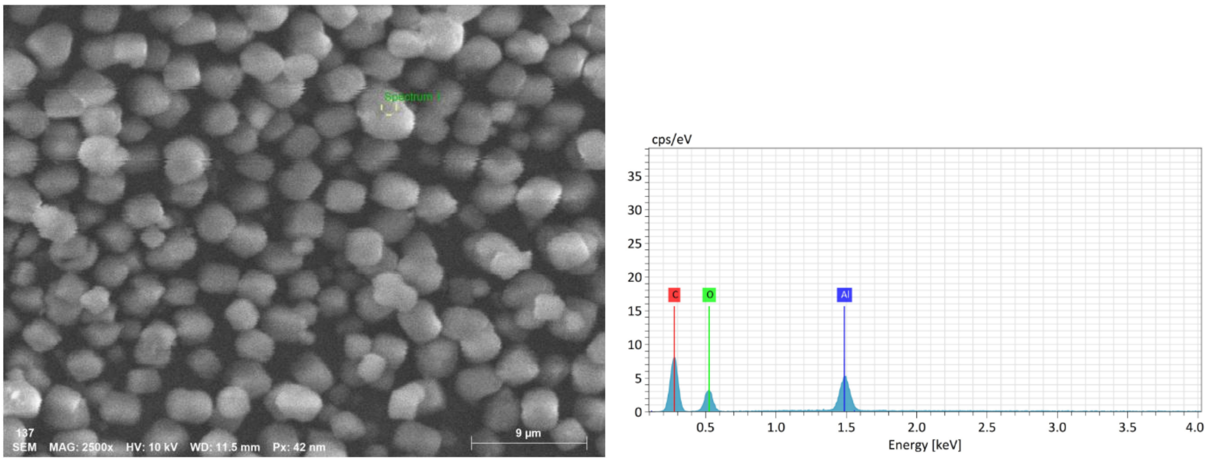

Figure S8: left: SEM micrograph of a sample of R-750 with the marked spot for EDX analysis. **right:** EDX spectrum of R-750.

SLS Analysis The particle size analysis was carried out in distilled water, one time by direct addition of CAU-10-H powder to the measurement chamber and 1 min of sonication within the device. Another measurement was done by preparing a dispersion of CAU-10-H in 15 mL of distilled water, sonication for 10 min, addition to the measurement chamber and sonication for 1 min within the device. The average of both values was calculated and plotted as the particle size distribution (PSD) of R-750 in **Figure S9**. The PSD of R-750 is slightly broader than that of the reference R-10. The error bars show quite a large deviation between the measurement after 1 min sonication versus the one after 11 min sonication for R-10, indicating a higher degree of agglomeration of particles in the products. Overall, the PSD of the large-scale synthesis product matches with the expected range from small-scale synthesis.

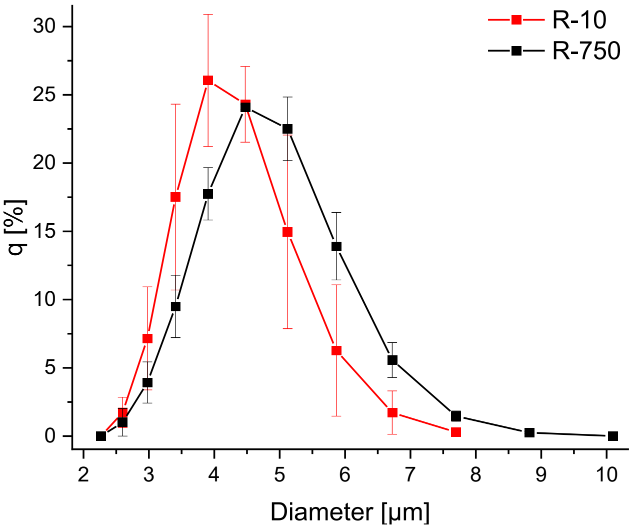

Figure S9: PSD of R-750 (black) compared to PSD of R-10 (red).

MIR spectroscopy The MIR spectra of R-750 and the reference batch R-10 are shown in **Figure S10**. The broad band from 3600 – 3100 cm^{-1} corresponds to the asymmetric and symmetric H-O-H stretching vibration of adsorbed water molecules. The marked bands at 3622 and 3685 cm^{-1} (*1) can be assigned to the μ_2-OH stretching vibration, which shift to higher wavenumbers upon the adsorption of water. The band at 3076 cm^{-1} (*2) and 1608 cm^{-1} (*3) can be assigned to the C-H and C=C vibrations of the aromatic ring of the linker. The spectral region from 1750 to 400 cm^{-1} of R-750 in comparison with pure isophthalic acid is shown in **Figure S10**. The bands at 1639, 1559 and 1428 cm^{-1} (*4, *5, *6, respectively) correspond to the stretching vibrational modes of the carboxylate groups, which are absent in the MIR-spectrum of the pure linker molecule.[51]

Band *1 proves the successful formation of the inorganic building unit and the bands *3, *5 and *6 prove full deprotonation of *m*-H_2BDC to *m*-BDC^{2-}. This indicates the coordination of aluminum ions by the linker and therefore the formation of the CAU-10-H framework. The band at 1130 cm^{-1} (*7) (**Figure S10 right**) can be assigned to the SO_4 stretching vibration of the sulfate ions and, together with the TG, EDX and elemental analysis, the sulfur content can be attributed to a neglectable amount of residual sulfate ions from the precursor most probably in the form of sodium sulfate.[51]

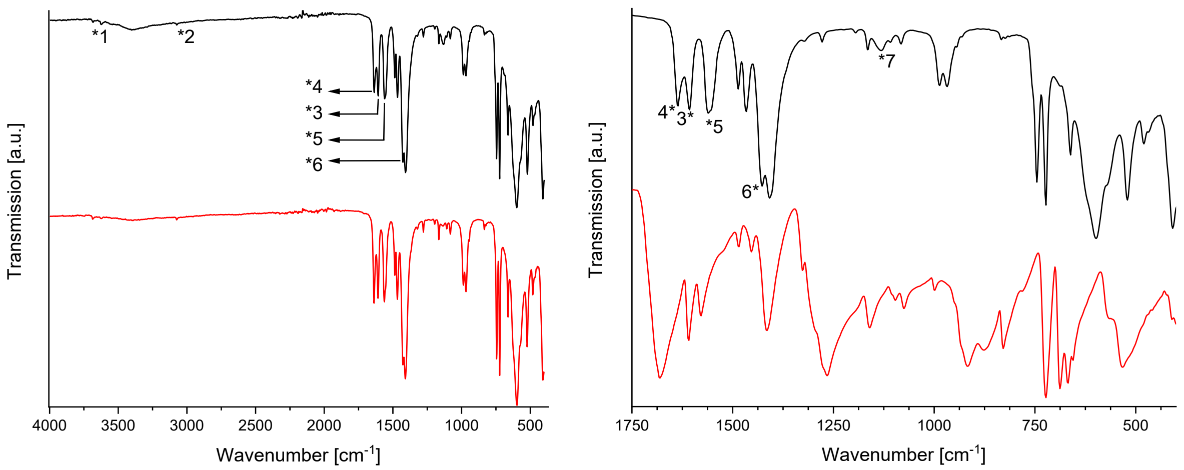

Figure S10: left: MIR spectrum of R-750 (black) with marked characteristic bands in comparison to R-10 (red). **right:** MIR spectrum of R-750 (black) with marked characteristic bands in comparison to *m*-H_2BDC (red).

Raman spectroscopy The Raman spectra of the scale-up product R-750 and the reference batch R-10 are shown in **Figure S11**.

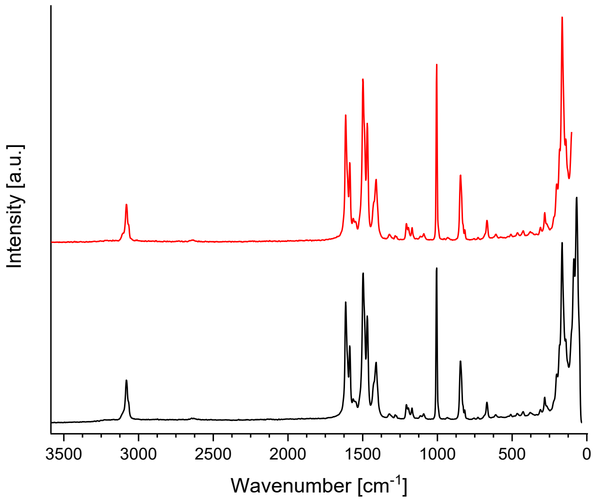

Figure S11: Raman spectra of R-750 (black) and the reference batches R-10 (red).

S4. Techno-economic analysis of CAU-10-H production costs

The simplified process flow diagram of the proposed industrial-scale production of CAU-10-H is represented in **Figure S12**. It is important to highlight that stainless steel (SS) 316 was selected as the material for the reactor, solution tanks, respective agitators and rotary dryer because, despite its high cost, it prevents potential short-term corrosion issues and extends equipment lifespan.

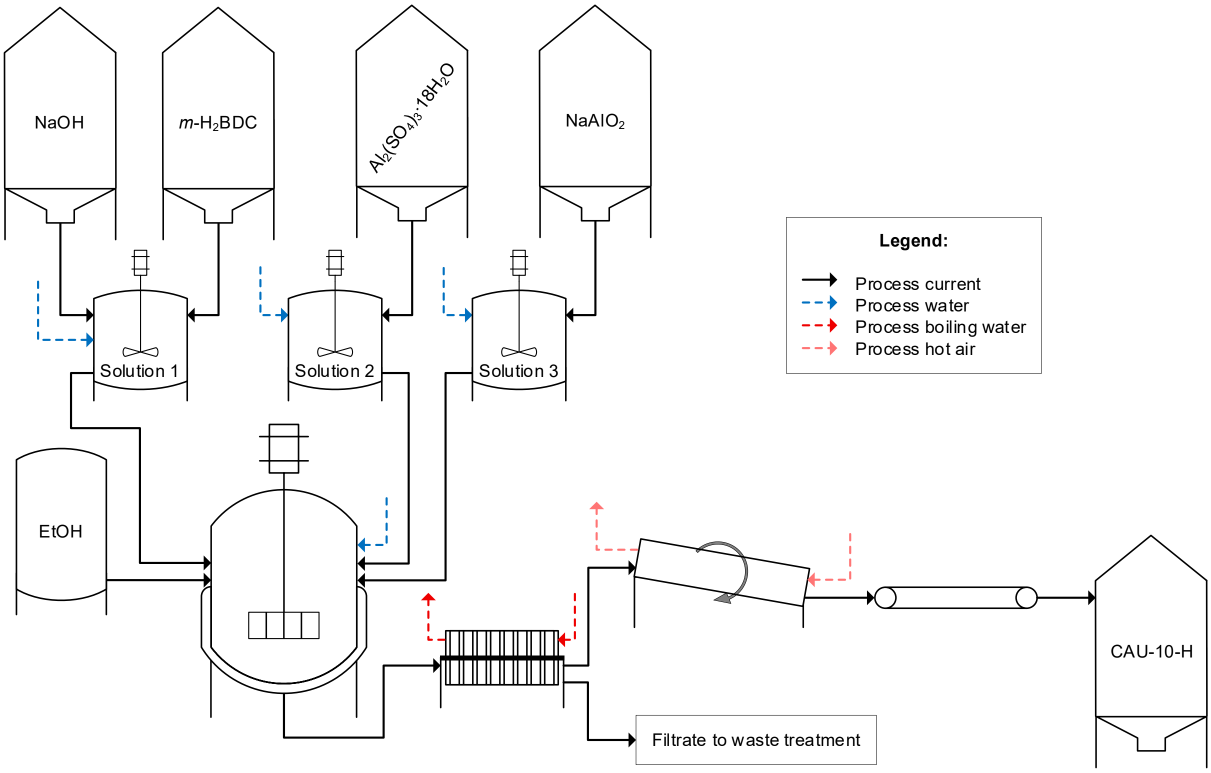

Figure S12: Process flow diagram for the industrial-scale production of CAU-10-H.

The cost of each equipment was obtained initially from databases indexed for the years 2014[52] and 2002[53], and later updated to the years 2019 and 2022 based on the Chemical Engineering Plant Cost Index (CEPCI), according to:

$$\text{Cost}_{\text{Year 2}} = \text{Cost}_{\text{Year 1}} \times \frac{\text{CEPCI}_{\text{Year 2}}}{\text{CEPCI}_{\text{Year 1}}} \quad \text{(S1)}$$

The final equipment costs and corresponding specifications used in the cost estimation of the first scenario are shown in **Table S8**. The storage bins and ethanol storage tank were dimensioned for 30 days of storage and with an extra 20 % of volume added, the storage/mixing tanks of solutions 1, 2 and 3 with an extra 15 % of volume, and the reactor was dimensioned to operate at 85 % of the full volume capacity.

Table S8: Major equipment cost for an STY of *ca*. 99 kg m^{-3} d^{-1}.

Major equipment	Relevant specifications	Cost 2019 / USD, k$	Cost 2022 / USD, k$	Ref.
Reactor + Agitator	$V_{reactor}$ = 78.4 m^3<br>$(L/D)_{reactor}$ = 1<br>$\delta_{reactor\ wall}$ = 0.005 m<br>$P_{agitator}$ = 15.7 kW<br>p_{atm}; SS-316<br>$E_{heat.T}$ = 22 440.9 MJ d^{-1}<br>$E_{maintain T}$ = 107.8 MJ d^{-1} [a]	894.6	1 201.7	52
Tanks + Agitators: Solutions 1, 2 & 3	V_{tank1} = 48.2 m^3<br>P_{tank1} = 9.6 kW<br>V_{tank2} = 20.2 m^3<br>P_{tank2} = 4.0 kW<br>V_{tank3} = 11.5 m^3<br>P_{tank3} = 2.3 kW<br>p_{atm}; SS-316	410.1	550.8	52
Storage: Isophthalic acid	ρ_{bulk} = 950 kg m^{-3} [b]<br>V_{bin} = 122.5 m^3<br>Carbon steel	36.9	49.6	52,54
Storage: Sodium hydroxide	ρ_{bulk} = 961 kg m^{-3}<br>V_{bin} = 58.4 m^3<br>Carbon steel	23.8	32.0	52,55
Storage: Ethanol	ρ = 786.4 kg m^{-3}<br>V_{tank} = 120.8 m^3<br>SS-304	129.2	173.5	52,56
Storage: Sodium aluminate	ρ_{bulk} = 977 kg m^{-3}<br>V_{bin} = 14.7 m^3<br>Carbon steel	14.6	19.5	52,55
Storage: Aluminum sulfate octadecahydrate	ρ_{bulk} = 820 kg m^{-3}<br>V_{bin} = 213.4 m^3<br>Carbon steel	55.4	74.4	52,57
Storage: CAU-10-H	ρ_{bulk} = 362.7 kg m^{-3} [c]<br>V_{bin} = 381.8 m^3<br>Carbon steel	88.8	119.3	52
Plate-and-frame filter	Dry/wet ratio = 43 % [c]<br>δ_{cake} = 0.05 m<br>L_{plate} = W_{plate} = 1.5 m<br>$A_{filtration}$ = 87.9 m^2 d^{-1}<br>SS-CF-8M<br>$m_{Water,boiler}$ = 36.4 t d^{-1} [d]<br>Boiler therm. eff. = 80 %<br>E_{boiler} = 15 229.7 MJ d^{-1}	237.2	318.6	53
Rotary dryer [e]	D = 0.46 m<br>L = 1.82 m<br>A_{drying} = 4.65 m^2<br>$P_{rotation}$ = 3.73 kW<br>Thermal eff. = 55 %<br>P_{drying} = 980.2 kW<br>SS-316	199.6	268.1	53
Conveyor belt	Speed = 1 m s^{-1}<br>W_{belt} = 0.4 m<br>L_{belt} = 50 m<br>$W_{solid\ transported}$ = 0.2 m<br>$H_{solid\ transported}$ = 0.05 m<br>P = 0.39 kW	89.4	120.1	53
**Total base equipment cost**		**2 179.5**	**2 927.6**	–

[a] Considering isolation with silica aerogel. [b] Average value as reported in reference [54]. [c] Experimental value. [d] Calculated from the washing water/dry product ratio from the values reported in **Sections 2.2** (280 kg of water) and **3.1** (29.6 kg of dry product as extrapolated in the main text). [e] A SS equipment for vacuum instead of a carbon steel one was assumed to avoid corrosion.

The main components of the fixed investment (FI) are the direct (D) and indirect (I) costs. The former can be estimated from the total cost of the base equipment (BE), whereas the latter can be estimated based on the direct costs. A risk provision (RP) to account for unforeseen events should also be considered. The risk provision is considered part of the indirect costs but we have opted to present it here separately for better clarity, with the total indirect costs given by ($Cost_I$+RP). Using common cost factor heuristics for chemical engineering processes[53] and the total base equipment cost listed in **Table S8**, the total fixed investment can be estimated according to:

$$FI = Cost_D + (Cost_I + RP) = \left[Cost_{BE} \times \sum_{n=1}^{9} f_n \times \left(1 + f_1^{'} + f_2^{'}\right)\right]\left(1 + f^{''}\right) \quad (S2)$$

with:

$$Cost_D = Cost_{BE} \times \sum_{n=1}^{9} f_n \quad (S3A)$$

$$Cost_I = Cost_D \times \left(f_1^{'} + f_2^{'}\right) \quad (S3B)$$

$$RP = (Cost_D + Cost_I) \times f^{''} \quad (S3C)$$

in which f_n represents the factors applied over the total base equipment cost in n different cost segments, $f_1^{'}$ and $f_2^{'}$ are, respectively, the factors applied over the direct cost for the project and supervision, and for the construction work, and $f^{''}$ is the factor for the risk provision, to be applied over the sum of all other direct and indirect costs.

The fixed investment, the working capital and the interim interests paid over the bank loan constitute the total investment. The working capital is considered as a percentage of the fixed investment. The interim interests consider that 60 % of the total interest-free investment will be obtained by bank loan, with a 5 % interest rate, and a plant construction period of 24 months during which only interim interests will be paid. The project time span is considered as 12 years, with the remaining 10 years for amortization consisting of plant operation. The estimation of the total investment for the first scenario is listed in **Table S9**.

Table S9: Segmentation of costs used to estimate the total investment for an STY of *ca*. 99 kg m^{-3} d^{-1}.

Cost segment	Factor, f_n	Cost 2019 / USD, k$	Cost 2022 / USD, k$
Base equipment (*cf*. **Table S8)**	1	**2 179.5**	**2 927.6**
Assembly of base equipment	0.45	980.8	1 317.4
Piping	0.30	653.9	878.3
Control	0.30	653.9	878.3
Buildings	0.15	326.9	439.1
Land and preparation	0.15	326.9	439.1
Electronic installations	0.15	326.9	439.1
Thermal isolation	0.10	218.0	292.8
Utilities and services:	0.112	244.1	327.9
Generated vapor	*0.03*	*65.4*	*87.8*
Vapor distribution	*0.01*	*21.8*	*29.3*
Substation electricity	*0.013*	*28.3*	*38.1*
Electricity distribution	*0.01*	*21.8*	*29.3*
Water for general use	*0.018*	*39.2*	*52.7*
Water distribution	*0.008*	*17.4*	*23.4*
Air of instrument	*0.01*	*21.8*	*29.3*
Effluent treatment	*0.013*	*28.3*	*38.1*
**Total direct costs ($Cost_D$)**		**5 910.9**	**7 939.6**

Cost segment	Factor, f_n'	Cost 2019/ USD, k$	Cost 2022/ USD, k$
Project and supervision	0.15	886.6	1 190.9
Construction work	0.15	886.6	1 190.9
**Indirect costs ($Cost_I$)**		**1 773.3**	**2 381.9**

Cost segment	Factor, f''	Cost 2019 / USD, k$	Cost 2022 / USD, k$
Provision for unforeseen events (RP)	0.15	1 152.6	1 548.2
**Total fixed investment (FI)**		**8 836.9**	**11 869.7**

	Factor	Cost 2019 / USD, k$	Cost 2022 / USD, k$
**Working capital**	0.20 (over total fixed investment)	**1 767.4**	**2 373.9**
**Total investment without interests**		10 604.2	14 243.7
**Interim interests**		676.9	909.2
**Total investment with interests**		**11 281.1**	**15 152.9**

The production costs are the sum of the manufacturing costs (with direct, indirect and fixed segments) and general expenses. The cost segmentation of each parcel for the first scenario is listed in **Table S11**. Where applicable, the currency values were converted to USD ($) using the average exchange rates reported for each year. The final production costs, presented in $ kg^{-1}, correspond to the total production costs divided by the yearly production of CAU-10-H of 1 kt. An uncertainty of ± 20 % (a common value in these estimates)[53] has also been included

in the final estimated production costs. The majority of cost segments were estimated using cost factors[53]. The exceptions are as follows:

- Total cost of raw materials, calculated for the yearly production and yield, based on the prices reported in **Table S10**, and updated to the years 2019 and 2022, as done for the equipment cost, but based on the *Producer prices in industry* (PPI) index as reported in the *Eurostat Data Browser*, with the following options;[58]
    - *Unit of measure – Index, 2021=100*
    - *Time frequency – Annual*
    - *Seasonal adjustment – Unadjusted data (i.e. neither seasonally adjusted nor calendar adjusted data)*
    - For all raw materials excluding water:
        - *Geopolitical entity – EU27_2020 (European Union - 27 countries (from 2020))*
        - *Statistical classification of economic activities in the European Community (NACE Rev. 2) – C20 (Manufacture of chemicals and chemical products)*
    - For water:
        - *Geopolitical entity – Germany; Finland*
        - *Statistical classification of economic activities in the European Community (NACE Rev. 2) – E36 (Water collection, treatment and supply)*

    For water, the corresponding PPI data was not available in the *EU27_2020 Geopolitical entity* for the year 1996 (base industrial water price value considered, *cf*. **Table S10**), so the average value of the available *Geopolitical entities* (Germany and Finland) was used as an approximation.
- Total annual operating labor cost, 32 047 € worker^{-1}, calculated for 2022 from the *Eurostat Data Browser* with the following options:[59]
    - *Age class – Total*
    - *Geopolitical entity – EU27_2020 (European Union - 27 countries (from 2020))*
    - *ISCO-08 code – OC-8 (Plant and machine operators and assemblers)*
    - *Sex – Total*
    - *Size classes in number of employees – GE10 (10 employees or more)*
    - *Structure of earnings indicator – ERN (Gross earnings); BNS (Annual bonuses)*

  The operating labor force considered consists of 25 persons operating the plant in 5 shifts (*i.e.*, 5 workers/shift). The operating labor cost for 2019 was retro-adjusted for inflation.[60]
- Utilities, considered as the boiling water used in the washing step (based on the 280 kg of boiling water used in R-750, *cf*. note **d** in **Table S8**) and the electricity requirements, calculated from the energy tariffs for 2019 (0.0506 € kWh^{-1}) and 2022 (0.1472 € kWh^{-1}), as listed in the *Eurostat Data Browser*[61] with the following options:
    - *Components of energy prices – NRG_SUP (Energy and supply)*
    - *Energy consumption – MWH2000-19999 (Consumption from 2 000 MWh to 19 999 MWh – band ID)*
    - *Geopolitical entity – EU27_2020 (European Union - 27 countries (from 2020))*

  The electricity considered corresponds to the energy required for the operation of the equipment pieces listed in **Table S8** (where applicable), to heat the reaction mixture in the reactor and maintain its temperature, and to heat the washing water in a boiler. Additionally, a 20 % overestimation in the total operation energy requirements is also

considered. The operation time of each equipment corresponds to the values reported in **Table S6** for R-750 for the first scenario, with the exception of the drying stage (considered as 6 h), and for R-750i for the second scenario.

- Depreciation costs, estimated from the fixed investment (**Table S9**) with the following depreciation rates:
  - 3 years for base equipment assembly, project and supervision;
  - 10 years for base equipment, piping, control, electronic installations, thermal isolation, utilities and services;
  - 25 years for buildings and construction work.
- Financial charges, taken as the average value of the yearly estimation from the capital borrowed from the bank considering the loan details described earlier.

Table S10: Prices considered for the raw materials used in the production of CAU-10-H.

Raw material	Price [a]	Notes
Isophthalic acid	1.84 $ kg^{-1}	*UN Comtrade Database*,[62] HS commodity code 291739 [b]
Aluminum sulfate	0.31 $ kg^{-1}	*UN Comtrade Database*,[62] HS commodity code 283322 [b]
Sodium aluminate	0.58 $ kg^{-1}	Average value of range in [63] and converted to $\$_{2015}$ kg^{-1}
Water (industrial)	1.71 $ m^{-3}	Average value of range in [64] and converted to $\$_{1996}$ m^{-3}
Sodium hydroxide	0.59 $ kg^{-1}	*Eurostat PRODCOM*,[65] PRRCODE 20132525 [c]
Ethanol	1.13 $ kg^{-1}	*UN Comtrade Database*,[62] HS commodity code 220720 [b,d]

[a] Values for the years reported. All prices were later updated to the years 2019 and 2022 based on the PPI index.

[b] Search conducted with the options *Reporter – European Union*; *Trade Flows – Import*; *Period – 2021*; *Partners – World*, and price calculated by dividing the import trade value by the import quantity.

[c] Search conducted with the options *DECL – EU27TOTALS_2020*; INDICATORS – *PRODQNT*, *PRODVAL*, *QNTUNIT*; *TIME_PERIOD – 2021*, and price calculated by dividing the sold production value (*PRODVAL*) by the sold production quantity (*PRODQNT*).

[d] Converted from $ L^{-1} with the density reported in [56].

Table S11: Segmentation of costs to estimate the production costs for an STY of *ca*. 99 kg m^{-3} d^{-1}.

Cost segment	Factor	Cost 2019 / USD, k$	Cost 2022 / USD, k$
Raw materials	–	2 799.6	3 873.7
Operating labor		800.1	844.3
Operating supervision	0.15 (over operating labor)	120.0	126.6
Utilities	–	324.7	845.5
Maintenance and repairs	0.07 (over total fixed investment)	618.6	830.9
Operating supplies	0.15 (over maintenance and repairs)	92.8	124.6
Laboratory charges	0.15 (over operating labor)	120.0	126.6
Royalties	0.04 (over manufacturing cost)	298.4	403.5
	**Direct costs**	**5 174.2**	**7 175.8**

Cost segment	Factor	Cost 2019 / USD, k$	Cost 2022 / USD, k$
**Indirect costs**	0.6 (over total labor and maintenance costs)	**923.2**	**1 081.1**

Cost segment	Factor	Cost 2019 / USD, k$	Cost 2022 / USD, k$
Depreciation	–	1 098.7	1 475.7
Taxes	0.02 (over total fixed investment)	176.7	237.4
Insurance	0.01 (over total fixed investment)	88.4	118.7
	**Fixed costs**	**1 363.8**	**1 831.8**
	**Manufacturing costs**	**7 461.1**	**$10 088.7**

Cost segment	Factor	Cost 2019 / USD, k$	Cost 2022 / USD, k$
Administration	0.6 (over operating labor)	480.0	506.6
Marketing, sales and distribution	0.2 (over production cost)	2 074.6	2 768.7
Financial charges	–	357.1	479.6
	**General expenses**	**2 911.7**	**3 754.9**

**Production cost (for a scale of 1 kt $year^{-1}$)**	10 372.8	13 843.6

**Final production cost ($ kg^{-1})[a]**	**10.4 ± 2.1**	**13.8 ± 2.8**

[a] Including an uncertainty of ± 20 %[53] in the final production cost.

For the second scenario, with an STY of *ca*. 481 kg m^{-3} d^{-1}, the differences in base equipment are the reactor, the tanks for solutions 1, 2 and 3 and the corresponding agitators, the storage bins for the reactants and ethanol tank, and the dryer, that have different dimensions than those of the first scenario, as listed in **Table S12**. The estimation of the investment and production costs for the higher STY are listed, respectively, in **Table S13** and **S14**.

Table S12: Major equipment cost for an STY of *ca*. 481 kg m^{-3} d^{-1} (excluding equipment equal to the first scenario, *cf*. **Table S8**).

Major equipment	Relevant specifications	Cost 2019 / USD, k$	Cost 2022 / USD, k$	Source
Reactor + Agitator	$V_{reactor}$ = 37.6 m^3<br>$(L/D)_{reactor}$ = 1<br>$\delta_{reactor\ wall}$ = 0.005 m<br>$P_{agitator}$ = 7.5 kW<br>p_{atm}; SS-316<br>$E_{heat.T}$ = 12 150.8 MJ d^{-1}<br>$E_{maintainT}$ = 28.3 MJ d^{-1} [a]	606.8	815.0	[52]
Tanks + Agitators: Solutions 1, 2 & 3	V_{tank1} = 24.6 m^3<br>P_{tank1} = 4.9 kW<br>V_{tank2} = 11.2 m^3<br>P_{tank2} = 2.2 kW<br>V_{tank3} = 5.7 m^3<br>P_{tank3} = 1.1 kW<br>p_{atm}; SS-316	344.8	463.2	[52]
Storage: Isophthalic acid	ρ_{bulk} = 950 kg m^{-3} [b]<br>V_{bin} = 117.6 m^3<br>Carbon steel	36.0	48.3	[52,54]
Storage: Sodium hydroxide	ρ_{bulk} = 961 kg m^{-3}<br>V_{bin} = 56.0 m^3<br>Carbon steel	23.3	31.3	[52,55]
Storage: Ethanol	ρ = 786.4 kg m^{-3}<br>V_{tank} = 57.4 m^3<br>SS-304	79.7	107.1	[52,56]
Storage: Sodium aluminate	ρ_{bulk} = 977 kg m^{-3}<br>V_{bin} = 14.1 m^3<br>Carbon steel	14.4	19.4	[52,55]
Storage: Aluminum sulfate octadecahydrate	ρ_{bulk} = 820 kg m^{-3}<br>V_{bin} = 205.0 m^3<br>Carbon steel	53.7	72.1	[52,57]
Rotary dryer [c]	D = 0.56 m<br>L = 2.23 m<br>A_{drying} = 6.51 m^2<br>$P_{rotation}$ = 5.59 kW<br>Thermal eff. = 55 %<br>P_{drying} = 1 470.2 kW<br>SS-316	276.4	371.3	[53]
	**Total base equipment cost [d]**	**1 850.5**	**2 485.5**	–

[a] Considering isolation with silica aerogel.

[b] Average value as reported in reference [54].

[c] A SS equipment for vacuum instead of a carbon steel one was assumed to avoid corrosion.

[d] Including storage bin for CAU-10-H, plate-and-frame filter and conveyor belt as dimensioned for the first scenario (*cf*. **Table S8**).

Table S13: Segmentation of costs used to estimate the total investment for an STY of *ca*. 481 kg m^{-3} d^{-1}.

Cost segment	Factor, f_n	Cost 2019 / USD, k$	Cost 2022 / USD, k$
Base equipment (*cf.* **Table S12**)	1	**1 850.5**	**2 485.5**
Assembly of base equipment	0.45	832.7	1 118.5
Piping	0.30	555.1	745.7
Control	0.30	555.1	745.7
Buildings	0.15	277.6	372.8
Land and preparation	0.15	277.6	372.8
Electronic installations	0.15	277.6	372.8
Thermal isolation	0.10	185.0	248.6
Utilities and services:	0.112	207.3	278.4
Generated vapor	*0.03*	*55.5*	*74.6*
Vapor distribution	*0.01*	*18.5*	*24.9*
Substation electricity	*0.013*	*24.1*	*32.3*
Electricity distribution	*0.01*	*18.5*	*24.9*
Water for general use	*0.018*	*33.3*	*44.7*
Water distribution	*0.008*	*14.8*	*19.9*
Air of instrument	*0.01*	*18.5*	*24.9*
Effluent treatment	*0.013*	*24.1*	*32.3*
**Total direct costs ($Cost_D$)**		**5 018.4**	**6 740.8**

Cost segment	Factor, f_n'	Cost 2019/ USD, k$	Cost 2022/ USD, k$
Project and supervision	0.15	752.8	1 011.1
Construction work	0.15	752.8	1 011.1
**Indirect costs ($Cost_D$)**		**1 505.5**	**2 022.2**

Cost segment	Factor, f''	Cost 2019 / USD, k$	Cost 2022 / USD, k$
Provision for unforeseen events (RP)	0.15	978.6	1 314.5
**Total fixed investment (FI)**		**7 502.6**	**10 077.5**
	**Factor**	**Cost 2019 / USD, k$**	**Cost 2022 / USD, k$**
**Working capital**	0.20 (over total fixed investment)	**1 500.5**	**2 015.5**
**Total investment without interests**		9 003.1	12 093.0
**Interim interests**		574.7	771.9
**Total investment with interests**		**9 577.7**	**12 864.9**

Table S14: Segmentation of costs to estimate the production costs for an STY of *ca*. 481 kg m^{-3} d^{-1}.

Cost segment	Factor	Cost 2019 / USD, k$	Cost 2022 / USD, k$
Raw materials	–	2 325.2	3 222.1
Operating labor	–	800.1	844.3
Operating supervision	0.15 (over operating labor)	120.0	126.6
Utilities	–	267.1	687.8
Maintenance and repairs	0.07 (over total fixed investment)	525.2	705.4
Operating supplies	0.15 (over maintenance and repairs)	78.8	105.8
Laboratory charges	0.15 (over operating labor)	120.0	126.6
Royalties	0.04 (over manufacturing cost)	260.9	349.2
	**Direct cost**	**4 497.2**	**6 167.9**

Cost segment	Factor	Cost 2019 / USD, k$	Cost 2022 / USD, k$
**Indirect cost**	0.6 (over total labor and maintenance costs)	**867.2**	**1 005.8**

Cost segment	Factor	Cost 2019 / USD, k$	Cost 2022 / USD, k$
Depreciation	–	932.8	1 252.9
Taxes	0.02 (over total fixed investment)	150.1	201.6
Insurance	0.01 (over total fixed investment)	75.0	100.8
	**Fixed cost**	**1 157.8**	**1 555.2**
	**Manufacturing cost**	**6 522.2**	**8 728.9**

Cost segment	Factor	Cost 2019 / USD, k$	Cost 2022 / USD, k$
Administration	0.6 (over operating labor)	480.0	506.6
Marketing, sales and distribution	0.2 (over production cost)	1 826.3	2 410.7
Financial charges	–	303.2	407.2
	**General expenses**	**2 609.5**	**3 324.4**

	**Production cost (for a scale of 1 kt year^{-1})**	**9 131.7**	**12 053.3**
	**Final production cost ($ kg^{-1})[a]**	**9.1 ± 1.8**	**12.1 ± 2.4**

[a] Including an uncertainty of ± 20 %[53] in the final production cost.

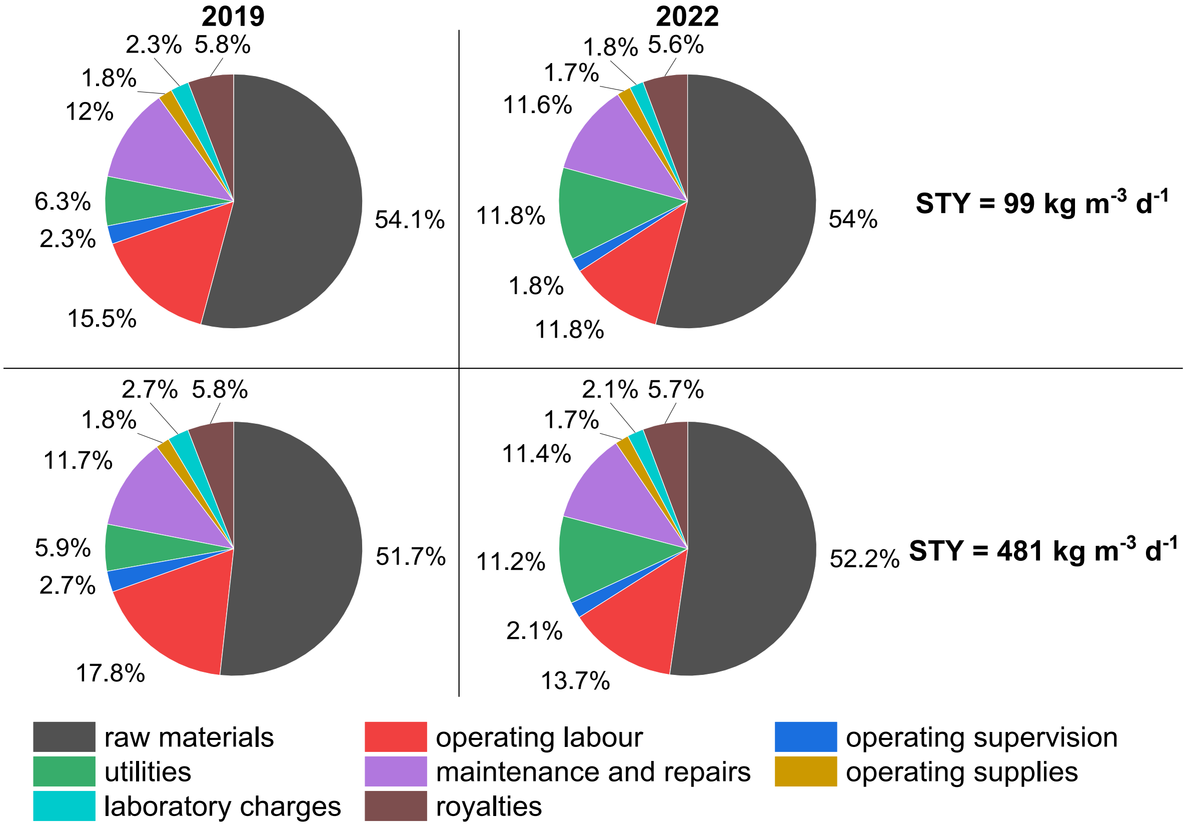

Figure S13: Representation of the individual cost segments of the direct costs of the manufacturing costs in percentages for STY's of *ca*. 99 kg m^{-3} d^{-1} and *ca*. 481 kg m^{-3} d^{-1} based on the available price information from 2019 in comparison with 2022.

S5. Further process optimization

For further process optimization, lab scale experiments were carried out with the aim to maximize the STY and overall efficiency. Using a 10 L glass reactor with a heating jacket the influence of recycling the solvent and increased synthesis concentration were investigated. The synthesis conditions for the recycling experiments were based on the molar ratios described in **Table S5**. The synthesis was stirred at 300 rpm at reflux conditions for a total of 14.6 h. The filtrate of the product mixture was recovered, cooled to ambient temperature, separated from the formed precipitate and used again for the next synthesis. For the experiments with increased synthesis concentration, the same conditions were used except the concentration of the starting solutions was doubled to 1 mol L^{-1}. In case that the minimum reaction time of 6 h is employed, the STY under these synthesis conditions will result in >400 kg m^{-3} d^{-1}. The characterization of the obtained products from the recycling experiments R-10-R0 to R-10-R3 and the product of a synthesis with increased concentration R-10-IC1 is given in the following section.

Characterization of CAU-10-H product batches with recycled solvent

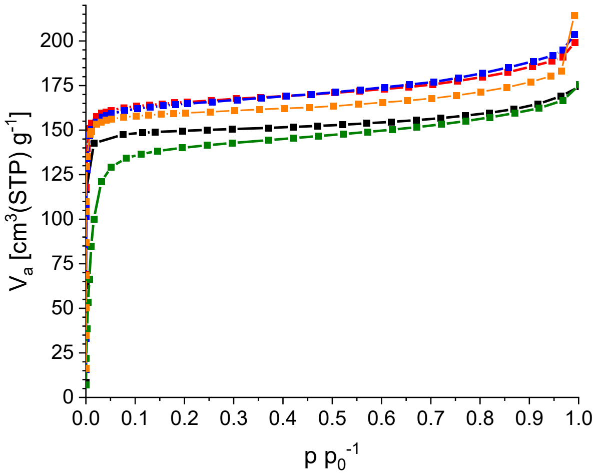

Figure S14: left: N_2 sorption isotherms of R-10-R0 (black), R-10-R1 (red), R-10-R2 (blue), R-10-R3 (green) and R-10-IC1 (orange) recorded at 77 K. Desorption branches are omitted for clarity.

Table S15: BET analysis results for CAU-10-H from syntheses with fresh solvent, one time recycled solvent, two times recycled solvent, three times recycled solvent and increased synthesis concentration.

**Sample**	A_{BET} **[m^2 g^{-1}]**	V_{mic} **[cm^3 g^{-1}]** (@p p_0^{-1} = 0.5)
R-10-R0	610	0.2362
R-10-R1	673	0.2643
R-10-R2	665	0.2647
R-10-R3	536	0.2278
R-10-IC1	656	0.2528

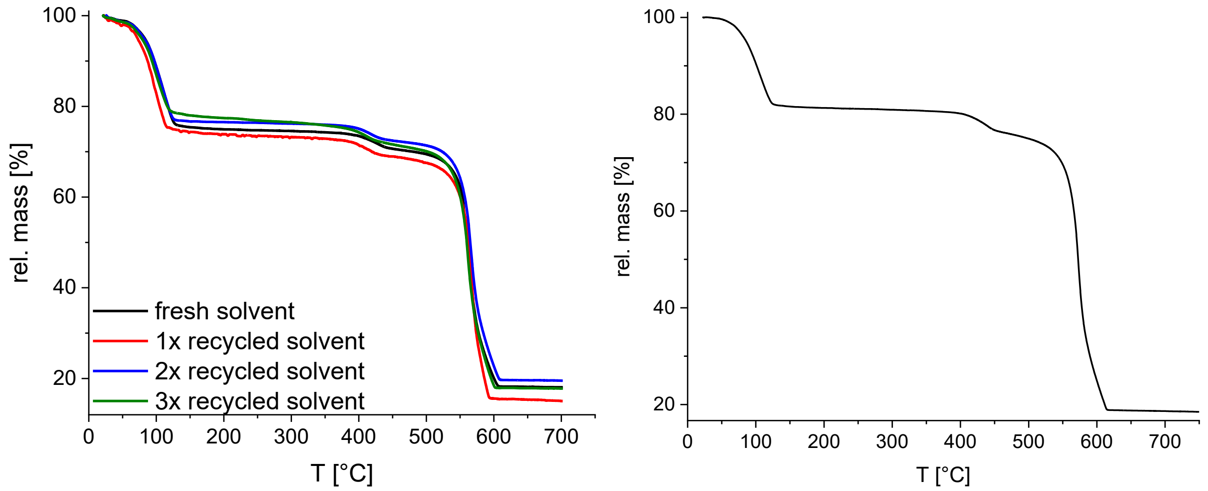

Figure S15: left: TG curves of CAU-10-H from syntheses with fresh solvent (black), one time recycled solvent (red), two times recycled solvent (blue) and three times recycled solvent (green). **right:** TG curve of 10 L laboratory-scale synthesis product R-10-IC1 (black) from synthesis with concentration of 1 mol L^{-1}.

Table S16: Observed and calculated mass loss steps of the TG data and observed and calculated values for the elemental analysis results of R-10-R0, R-10-R1, R-10-R2, R-10-R3 and R-10-IC1.

**Sample**	**R-10-R0**		**R-10-R1**		**R-10-R2**		**R-10-R3**		**R-10-IC1**	
Sum formula	$[Al(OH)(C_8H_4O_4)] \cdot 4\ H_2O$		$[Al(OH)(C_8H_4O_4)] \cdot 4.6\ H_2O \cdot 0.2\ Na_2BDC$		$[Al(OH)(C_8H_4O_4)] \cdot 3.6\ H_2O$		$[Al(OH)(C_8H_4O_4)] \cdot 3.5\ H_2O$		$[Al(OH)(C_8H_4O_4)] \cdot 1.35\ H_2O \cdot 0.2\ Na_2BDC$	
**TGA**	Obs.	Calc.	Obs.	Calc.	Obs.	Calc.	Obs.	Calc.	Obs.	Calc.
Solvent step [%] T = 20 – 300 °C	25.44	25.47	26.77	26.77	23.80	23.84	23.49	22.31	19.08	19.07
Framework step [%] T = 300 – 700 °C	56.48	56.51	58.06	58.09	56.64	57.50	58.68	58.66	62.27	62.36
**Elemental Analysis**										
C ± 0.06 [%]	34.47	34.53	34.49	34.71	35.07	35.16	35.90	35.87	42.2	42.01
H ± 0.05 [%]	4.49	4.67	4.43	4.52	4.40	4.51	4.16	4.38	3.30	3.12
N ± 0.14 [%]	0	0	0	0	0	0	0	0	0	0
S ± 0.08 [%]	0	0	0	0	0	0	0	0	0	0

S6. Evaluation of CAU-10-H for ADC

Electricity saving potential of ADC devices The comparison of electric power consumption of conventional compression cooling and adsorption cooling must cover the functional levels of:

A) the basic reverse heat pumping ("cold generation"),

B) the transport of thermofluids inside the chillers by pumps,

C) the recooling outside the chillers (pumps and fans).

As the cold distribution into and in the target building or process as well as the small demand for process control is independent from the type of cold generation, it is the same for both compression and adsorption cooling and does not need to be considered for the comparison. Including dry air-recooling, common commercial compression chillers do have electrical efficiency ratios (EER) of about 2.5 to 4, whereas adsorption chillers with recoolers reach EER of 12 – 20. Following the equation:

$$Electricity\ saving = \frac{1 - EER_{comp}}{EER_{Ads}} \times 100 \tag{S4}$$

adsorption-based cooling saves 66 – 87 % of electric power for cooling.[66–68] For both technologies, real data is strongly influenced by individual system installations (*e.g.*, length and diameter of pipes), operational parameters and local climate conditions.

Expected CAU-10-H demand for ADC Based on the data in this study a cooling power of 0.88 kW kg^{-1} CAU-10-H for a state-of-the-art ADC (at BAFA reference conditions HT: 85 °C/MT: 27 °C/LT: 19 °C) can be expected. Correlation with a conservative 2027 adsorption cooling market perspective of new 2 MW cooling power installation (sufficient for around 30 000 m² air conditioning) leads to a need of around 2 500 kg CAU-10-H.

Preparation of CAU-10-H coatings For the evaluation of the performance of shaped CAU-10-H for its application in ADC units, several 50 × 50 × 1 mm aluminum plates were prepared at the Kiel laboratories and sent to the SorCool facility for measurement. The preparation was based on the published protocol of Lenzen *et al.* and further adapted to yield high quality samples.[41] The aluminum plates were pretreated with diluted HCl, cleaned with acetone and rinsed with distilled water. The coated area was limited by using adhesive strips and covering the sites of the plate in accordingly folded aluminum foil to prevent the slurry from flowing off the plates. The coating slurry was prepared by dispersing 1 g CAU-10-H in 2.316 g distilled water by supersonic treatment. Subsequently, 319 mg of SilRes MP50E binder was added under stirring to yield the final coating slurry. The plates were placed in a petri dish and homogeneously coated with the slurry using a pipette. The coated aluminum plates were dried overnight under ambient conditions. The thickness of the coatings was determined as the average out of 4 points measured with a digital caliper. For the samples C1 and C2 the average thickness was determined as 260 µm and 340 µm, respectively.

Sorption kinetics measurement In contrast to the described equilibrium measurements, kinetic measurements were conducted by using the vapor lines via V7 instead of V6 during the adsorption step. After stabilizing temperature and water vapor pressure, V7 was opened to let the water vapor flow into the measurement chamber. The mass flow was recorded using two mass flow sensors F-1 and F-2 (Vögtlin). For comparison of different samples, the mass flow

rates (g s^{-1}) are scaled to the active (dry) mass of adsorbent (g g^{-1} s^{-1}) or the coated surface (g g^{-1} m^{-2}).

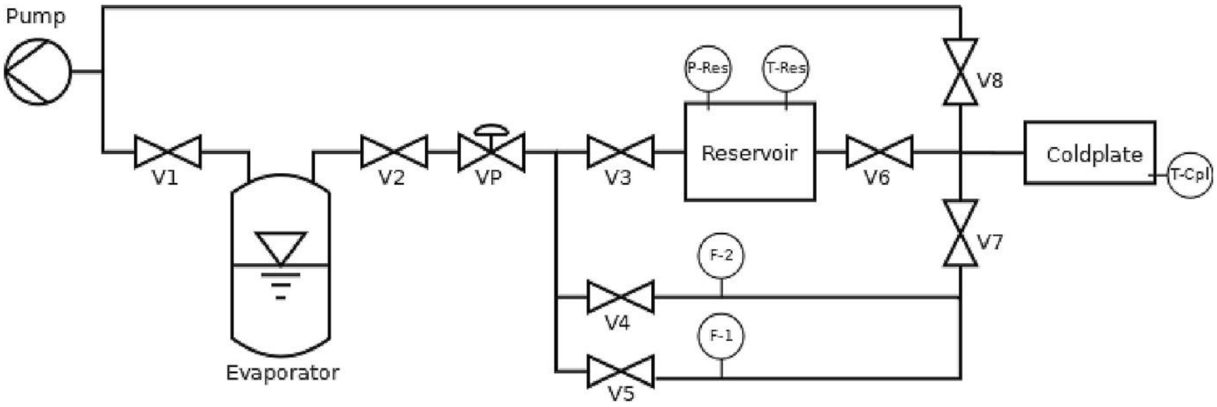

Figure S16: P&ID scheme of the measurement set up used for the evaluation of CAU-10-H coated aluminum plates for their application in ADC units. The figure was reproduced with permission by the authors.[69]

The estimation of CAU-10-H performance in a given adsorption chiller was carried out via a bottom-up simulation of this machine running with silica gel or SAPO-34 as the adsorbent - based on full measurements on existing machines - with the active adsorbent layers on the heat exchanger fins substituted by CAU-10-H layers on same fins.

The mass transport outside the adsorbent layer is determined by the construction of the adsorption module containing evaporator and adsorber heat exchanger. The heat transport is determined by the construction of the adsorber heat exchanger, both showing no limits even for the higher heat of adsorption and the water capacity of SAPO-34. As the simulation is based on the identical construction, it can be concluded that there is no (or no larger than already applied for silica gel or SAPO-34 simulation) limitation of CAU-10-H performance. In that way, the measured kinetic and equilibrium data (**Figure 6** in the main manuscript) can directly be used to estimate the machine performance.

We are aware that it is important to avoid mass and heat transfer limitations as good as possible as they are limiting the performance of adsorption chillers.

Another factor of similar importance is the purity specification regarding synthesis and application. As adsorption systems run in vacuum modules with only the water vapor pressure, impurities generating additional gases always increase pressure and reduce performance significantly. Therefore, volatile impurities and remaining solvents (except water) must be excluded. The applied aqueous synthesis path with thorough drying is a very reliable method. Pore blocking by remaining reactants or side products must be avoided also. It is done in a satisfying amount by repeated washing of the MOF material as demonstrated by the samples in the equilibrium measurements reaching the expected full water capacity for CAU-10-H.

In a full technical scale, quality control measures must be implemented. In continuation of the presented investigations, long-term tests of CAU-10-H adsorption chillers are required to gain more answers on adsorbent impurity and particle size effects and on altering behavior.

S7. References

(1) Achenbach, B.; Yurdusen, A.; Stock, N.; Maurin, G.; Serre, C. Synthetic Aspects and Characterization Needs in MOF Chemistry – from Discovery to Applications. *Advanced Materials* **2025**, 2411359. https://doi.org/10.1002/adma.202411359.
(2) Sigma-Aldrich, Basolite® F300. . https://www.sigmaaldrich.com/DE/de/product/aldrich/690872 (accessed 2024-01-09).
(3) Sigma-Aldrich, Basolite® Z377. . https://www.sigmaaldrich.com/DE/de/product/aldrich/794325 (accessed 2025-03-26).
(4) Sigma-Aldrich, Basolite® Z1200. . https://www.sigmaaldrich.com/DE/de/product/aldrich/691348 (accessed 2025-03-26).
(5) Framergy, AYRSORB ™ F100. . https://framergy.com/AYRSORB-F100.php (accessed 2025-03-26).
(6) Framergy, AYRSORB ™ F250. . https://www.framergy.com/AYRSORB-F250.php (accessed 2025-03-26).
(7) Framergy, AYRSORB ™ F125. . https://www.framergy.com/AYRSORB-T125.php (accessed 2025-03-26).
(8) Framergy, AYRSORB ™ P151. . https://www.framergy.com/AYRSORB-P151.php (accessed 2025-03-26).
(9) Ascensus Specialties, STREM catalog, CAU-10. . https://www.strem.com/product/13-0300 (accessed 2025-03-26).
(10) Ascensus Specialties, STREM catalog, HKUST-1(Cu). . https://www.strem.com/product/29-3050 (accessed 2025-03-26).
(11) Ascensus Specialties, STREM catalog, MIL-53(Al). . https://www.strem.com/product/13-3050 (accessed 2024-01-09).
(12) Ascensus Specialties, STREM catalog, MIL-101(Al)-NH_2. . https://www.strem.com/product/13-3060 (accessed 2024-01-09).
(13) Ascensus Specialties, STREM catalog, UiO-66-BDC-COOH. . https://www.strem.com/product/40-1111 (accessed 2025-03-26).
(14) Ascensus Specialties, STREM catalog, UiO-66-FA. . https://www.strem.com/product/40-1114 (accessed 2025-03-26).
(15) Ascensus Specialties, STREM catalog, UiO-66(Zr). . https://www.strem.com/product/40-1105 (accessed 2025-03-26).
(16) Ascensus Specialties, STREM catalog, ZIF-8. . https://www.strem.com/product/30-4015 (accessed 2025-03-26).
(17) MOFapps AS, about MOFs. . http://www.mofapps.com/about-mofs/ (accessed 2025-03-26).
(18) Promethean Particles®, Metal-Organic Frameworks. . https://prometheanparticles.co.uk/metal-organic-frameworks-mofs/ (accessed 2025-03-26).
(19) ProfMOF AS, Our MOF products. . https://profmof.com/products/ (accessed 2025-03-26).
(20) Immaterial Ltd., Our focus. . https://immaterial.com/focus/ (accessed 2025-03-26).
(21) Mosaic Materials Inc., About. . https://mosaicmaterials.com/about/ (accessed 2025-03-26).
(22) NovoMOF AG, Services. . https://novomof.com/services (accessed 2025-03-26).
(23) MOF Technologies Ltd., Nuada, The next-generation carbon capture technology. . https://nuadaco2.com/technology/ (accessed 2025-03-26).
(24) Numat Technologies, Inc., ION-X®. . https://www.numat.com/solutions/ion-x/ (accessed 2025-03-26).
(25) Numat Technologies, Inc., Sentinel™. . https://www.numat.com/solutions/sentinel/ (accessed 2025-03-26).
(26) Svante Technologies Inc., carbon capture technology. . https://www.svanteinc.com/carbon-capture-technology/ (accessed 2025-03-26).
(27) SquairTech, Our technologies, ST-Sorb13. . https://www.squair.tech/sorb13.html (accessed 2025-03-26).

(28) SquairTech, Our technologies, ST-Cata26. . https://www.squair.tech/cata26.html (accessed 2025-03-26).
(29) Vector Bioscience Cambridge, The most versatile targeted delivery. . https://vectorbiocam.com (accessed 2025-03-26).
(30) Nanoshell-UK Ltd.; exemplary quote; personal communication. Quote from Nanoshell-UK Ltd., 2024.
(31) BLDPharm, Catalog, MIL-101(Al)-NH_2. . https://www.bldpharm.com/products/1404201-64-4.html (accessed 2025-03-26).
(32) Chiou, D.; Yu, H. J.; Hung, T.; Lyu, Q.; Chang, C.; Lee, J. S.; Lin, L.; Kang, D. Highly CO_2 Selective Metal–Organic Framework Membranes with Favorable Coulombic Effect. *Adv Funct Materials* **2021**, *31* (4), 2006924. https://doi.org/10.1002/adfm.202006924.
(33) Jin, H.; Wollbrink, A.; Yao, R.; Li, Y.; Caro, J.; Yang, W. A Novel CAU-10-H MOF Membrane for Hydrogen Separation under Hydrothermal Conditions. *Journal of Membrane Science* **2016**, *513*, 40–46. https://doi.org/10.1016/j.memsci.2016.04.017.
(34) Chiou, D.; Yu, H. J.; Hung, T.; Lyu, Q.; Chang, C.; Lee, J. S.; Lin, L.; Kang, D. Highly CO_2 Selective Metal–Organic Framework Membranes with Favorable Coulombic Effect. *Adv Funct Materials* **2021**, *31* (4), 2006924. https://doi.org/10.1002/adfm.202006924.
(35) Jin, H.; Wollbrink, A.; Yao, R.; Li, Y.; Caro, J.; Yang, W. A Novel CAU-10-H MOF Membrane for Hydrogen Separation under Hydrothermal Conditions. *Journal of Membrane Science* **2016**, *513*, 40–46. https://doi.org/10.1016/j.memsci.2016.04.017.
(36) Zhao, Y.; Gao, G.; Zhu, P.; Liu, H.; Guo, Y.; Zhang, X. Induced Synthesis of an Al-Based CAU-10 Tubular Membrane for the Highly Efficient Separation of MeOH/MTBE by Pervaporation. *Ind. Eng. Chem. Res.* **2023**, *62* (44), 18694–18703. https://doi.org/10.1021/acs.iecr.3c03138.
(37) Brandt, P.; Xing, S.-H.; Liang, J.; Kurt, G.; Nuhnen, A.; Weingart, O.; Janiak, C. Zirconium and Aluminum MOFs for Low-Pressure SO_2 Adsorption and Potential Separation: Elucidating the Effect of Small Pores and NH_2 Groups. *ACS Appl. Mater. Interfaces* **2021**, *13* (24), 29137–29149. https://doi.org/10.1021/acsami.1c06003.
(38) Ping, E.; Chen, X.; Zhou, Y.; Zhang, L.; Kong, L.; Chen, N. H_2/D_2 Separation Using UTSA-16@CAU-10-H@γ-AlOOH Composites as the Stationary Phase in Gas Chromatography via the Additive Effects of Kinetic Sieving and Chemical Affinity Quantum Sieving. *Inorg. Chem.* **2023**, *62* (4), 1591–1601. https://doi.org/10.1021/acs.inorgchem.2c03795.
(39) Xu, M.; Tang, W.-Q.; Meng, S.-S.; Gu, Z.-Y. Metal–Organic Frameworks for the Separation of Xylene Isomers. *Chem. Soc. Rev.* **2025**, *54* (3), 1613–1633. https://doi.org/10.1039/D4CS00796D.
(40) Pei, J.; Wen, H.; Gu, X.; Qian, Q.; Yang, Y.; Cui, Y.; Li, B.; Chen, B.; Qian, G. Dense Packing of Acetylene in a Stable and Low-Cost Metal–Organic Framework for Efficient C_2H_2/CO_2 Separation. *Angewandte Chemie* **2021**, *133* (47), 25272–25278. https://doi.org/10.1002/ange.202110820.
(41) Lenzen, D.; Bendix, P.; Reinsch, H.; Fröhlich, D.; Kummer, H.; Möllers, M.; Hügenell, P. P. C.; Gläser, R.; Henninger, S.; Stock, N. Scalable Green Synthesis and Full-Scale Test of the Metal–Organic Framework CAU-10-H for Use in Adsorption-Driven Chillers. *Advanced Materials* **2018**, *30* (6), 1705869. https://doi.org/10.1002/adma.201705869.
(42) Zheng, Z.; Alawadhi, A. H.; Yaghi, O. M. Green Synthesis and Scale-Up of MOFs for Water Harvesting from Air. *Mol. Front. J.* **2023**, *07* (01n02), 20–39. https://doi.org/10.1142/S2529732523400011.
(43) Seo, Y.-K.; Yoon, J. W.; Lee, J. S.; Lee, U.-H.; Hwang, Y. K.; Jun, C.-H.; Horcajada, P.; Serre, C.; Chang, J.-S. Large Scale Fluorine-Free Synthesis of Hierarchically Porous Iron(III) Trimesate MIL-100(Fe) with a Zeolite MTN Topology. *Microporous and Mesoporous Materials* **2012**, *157*, 137–145. https://doi.org/10.1016/j.micromeso.2012.02.027.

(44) Chebbi, M.; Azambre, B.; Volkringer, C.; Loiseau, T. Dynamic Sorption Properties of Metal-Organic Frameworks for the Capture of Methyl Iodide. *Microporous and Mesoporous Materials* **2018**, *259*, 244–254. https://doi.org/10.1016/j.micromeso.2017.10.018.
(45) Bae, H. J.; Kim, S.-I.; Choi, Y.; Kim, K.-M.; Bae, Y.-S. High P-Xylene Selectivity in Aluminum-Based Metal–Organic Framework with 1-D Channels. *Journal of Industrial and Engineering Chemistry* **2023**, *117*, 333–341. https://doi.org/10.1016/j.jiec.2022.10.021.
(46) Zhang, X.; Lin, R.-B.; Wu, H.; Huang, Y.; Ye, Y.; Duan, J.; Zhou, W.; Li, J.-R.; Chen, B. Maximizing Acetylene Packing Density for Highly Efficient C2H2/CO2 Separation through Immobilization of Amine Sites within a Prototype MOF. *Chemical Engineering Journal* **2022**, *431*, 134184. https://doi.org/10.1016/j.cej.2021.134184.
(47) Silva, M. P.; Ribeiro, A. M.; Silva, C. G.; Nogueira, I. B. R.; Cho, K.-H.; Lee, U.-H.; Faria, J. L.; Loureiro, J. L.; Chang, J.-S.; Rodrigues, A. E.; Ferreira, A. MIL-160(Al) MOF's Potential in Adsorptive Water Harvesting. *Adsorption* **2021**, *27* (2), 213–226. https://doi.org/10.1007/s10450-020-00286-5.
(48) Rouquerol, J.; Llewellyn, P.; Rouquerol, F. Is the Bet Equation Applicable to Microporous Adsorbents? In *Studies in Surface Science and Catalysis*; Elsevier, 2007; Vol. 160, pp 49–56. https://doi.org/10.1016/S0167-2991(07)80008-5.
(49) Röhrscheid, F. Carboxylic Acids, Aromatic. In *Ullmann's Encyclopedia of Industrial Chemistry*; Wiley-VCH, Ed.; Wiley, 2000. https://doi.org/10.1002/14356007.a05_249.
(50) Sigma-Aldrich, Safety Data Sheet Isophthalic acid (I19209). Safety Data Sheet Isophthalic Acid (I19209), 2024. https://www.sigmaaldrich.com/DE/en/sds/aldrich/i19209?userType=anonymous (accessed 2024-07-02).
(51) Socrates, G.; Socrates, G. *Infrared and Raman Characteristic Group Frequencies: Tables and Charts*, 3rd ed.; Wiley: Chichester ; New York, 2001.
(52) Milligan, D.; Milligan, J. *Matches' engineering to chemical energy manufacturing metallurgical industries*. https://www.matche.com/default.html (accessed 2025-11-09).
(53) Peters, M. S.; Timmerhaus, K. D.; West, R. E. *Plant Design and Economics for Chemical Engineers*; McGraw-Hill: New York, 2003.
(54) Indorama Ventures, Technical Data Sheet Isophthalic acid. Technical Data Sheet Isophthalic Acid, 2025. https://www.indoramaventures.com/storage/downloads/product/feedstock/pia/PIA-TDS-IVXP-USA.pdf.
(55) Anval Valves PVT Ltd., Bulk Density Chart. . https://anval.net/Downloads/Bulk%20Density%20Chart.pdf (accessed 2025-08-06).
(56) Friedrich Scharr KG, Safety Data Sheet; Spiritus 94 %. 2025. https://scharr.de/fluid/chemieprodukte/produktdatenbank/detail?tx_bbchemdox_detail%5Baction%5D=getdocument&tx_bbchemdox_detail%5Bcontroller%5D=Chemdox&tx_bbchemdox_detail%5Bid%5D=ID%2001670%20SDS-002&tx_bbchemdox_detail%5Bprefix%5D=Safety%20Data%20Sheet&tx_bbchemdox_detail%5Btitle%5D=Spiritus%2094%20%25-SDS%20-2024-06-21&cHash=4c5fa60c92334b631c35b07ecee12af3 (accessed 2025-07-18).
(57) ChemicalBook, CAS DataBase List, Aluminiumsulfate Octadecahydrate. . https://www.chemicalbook.com/ChemicalProductProperty_EN_CB5435191.htm (accessed 2025-08-06).
(58) Eurostat; Producer prices in industry, total - annual data. . https://ec.europa.eu/eurostat/databrowser/product/page/STS_INPP_A (accessed 2025-11-19).
(59) Eurostat, Mean annual earnings by sex, age and occupation - NACE Rev. 2, B-S excluding O (2022). . https://ec.europa.eu/eurostat/databrowser/product/page/EARN_SES22_28 (accessed 2025-08-06).

(60) The World Bank Group; Inflation, consumer prices (annual %) - European Union. . https://data.worldbank.org/indicator/FP.CPI.TOTL.ZG?end=2022&locations=EU&start=2019&view=chart (accessed 2025-07-18).
(61) Eurostat, Electricity prices components for non-household consumers - annual data (from 2007 onwards). . https://ec.europa.eu/eurostat/databrowser/view/nrg_pc_205_c/default/table?lang=en (accessed 2025-03-26).
(62) United Nations, UN Comtrade Database. . https://comtradeplus.un.org/ (accessed 2025-03-26).
(63) Ye, F.; He, B.; Tian, C.; Zhao, M.; Wang, J.; Han, X.; Armaghani, D. J. Influence of Sodium Aluminate on Calcium Leaching of Shotcrete in Tunnels. *Tunnelling and Underground Space Technology* **2021**, *117*, 104156. https://doi.org/10.1016/j.tust.2021.104156.
(64) European Environment Agency, Agricultural, industrial and household water prices in late 1990s. . https://www.eea.europa.eu/data-and-maps/figures/agricultural-industrial-and-household-water-prices-in-late-1990s (accessed 2025-07-18).
(65) Eurostat; Sold production, exports and imports.; PRCCODE: [20132525] Sodium Hydroxide (caustic soda), solid. . https://ec.europa.eu/eurostat/databrowser/view/ds-059358/legacyMultiFreq/table?lang=en (accessed 2025-07-18).
(66) Fahrenheit GmbH; Fahrenheit eCoo 10X Productinformation. . https://fahrenheit.cool/produkt/ecoo-10x/ (accessed 2025-09-01).
(67) International Energy Agency. *The Future of Cooling - Opportunities for Energy-Efficient Air Conditioning*; 2018. https://iea.blob.core.windows.net/assets/0bb45525-277f-4c9c-8d0c-9c0cb5e7d525/The_Future_of_Cooling.pdf (accessed 2025-09-10).
(68) Adsorbus GmbH; Adsorbus Homepage. . https://adsorbus.com/adsorptionskaelte/ (accessed 2025-09-01).
(69) Velte-Schäfer, A.; Zhang, Y.; Nonnen, T.; Wittstadt, U.; Frazzica, A.; Füldner, G.; Palomba, V. Numerical Modelling and Evaluation of a Novel Sorption Module for Thermally Driven Heat Pumps and Chillers Using Open-Source Simulation Library. *Energy Conversion and Management* **2023**, *291*, 117252. https://doi.org/10.1016/j.enconman.2023.117252.
